# Aptamer-based proteomics in pediatric patients with severe traumatic brain injury: a pilot study

**DOI:** 10.1038/s41390-026-04798-8

**Published:** 2026-02-19

**Authors:** Bradley J. De Souza, Michael S. Wolf, Jeffrey R. Leonard, Jinsheng Yu, Jose A. Pineda

**Affiliations:** 1 Department of Anesthesiology Critical Care Medicine, Children’s Hospital Los Angeles, Los Angeles, CA USA; 2 Division of Critical Care Medicine, Monroe Carell Jr. Children’s Hospital at Vanderbilt, Nashville, TN USA; 3 Department of Neurological Surgery, Nationwide Children’s Hospital, Columbus, OH USA; 4 Department of Genetics and McDonnell Genome Institute, Washington University in Saint Louis, St. Louis, MO USA; 5 Division of Critical Care Medicine, Department of Pediatrics, David Geffen School of Medicine at UCLA, Los Angeles, CA USA

## Abstract

**Background:**

Severe traumatic brain injury (sTBI) in children is a major cause of morbidity and mortality, yet its biology and prognostic indicators remain partially defined. Aptamer-based proteomics enables high-throughput characterization of molecular responses and may reveal biomarkers that improve injury characterization and outcome prediction.

**Methods:**

In this prospective, repeated-measures case-control pilot study, we characterized temporal changes in the plasma proteome after pediatric sTBI and evaluated their relationship to short-term outcomes. Plasma samples from children with sTBI (*n* = 24) were collected at 24- and 72-hours after injury and compared with healthy controls (*n* = 4). Samples were analyzed using an aptamer-based assay measuring 1297 proteins. Principal component analysis, differential expression testing, and pathway enrichment were performed. Associations with Glasgow Outcome Scale – Extended, Pediatric Revision (GOS–E Peds) scores at hospital discharge and 3-months were examined.

**Results:**

634 proteins showed significant temporal changes, including established and novel biomarkers. Proteomic profiles diverged from controls, greatest at 72 hours. Age accounted for substantial variability within sTBI patients. Distinct protein panels predicted GOS–E Peds outcomes at discharge (R^2^ = 0.84) and 3-months (R^2^ = 0.88).

**Conclusion:**

Temporally resolved plasma proteomics may identify evolving brain and systemic responses after pediatric sTBI that are associated with short-term outcomes.

**Impact:**

- Temporally resolved aptamer-based plasma proteomics reveal dynamic shifts in brain-specific and systemic signaling after pediatric severe traumatic brain injury (sTBI) and correlate with short-term neurologic outcomes.
- We identify 634 proteins with significant temporal changes, including both established and novel biomarkers, and show that age is a major determinant of proteomic variability in patients with sTBI.
- This study demonstrates the feasibility and clinical potential of high-dimensional proteomic profiling for biomarker discovery, molecular phenotyping, and potential for outcome prediction, informing future precision medicine approaches in pediatric neurocritical care.

## Introduction

Severe traumatic brain injury (sTBI) remains a leading cause of mortality and long-term disability in children, yet its complex pathobiology and mechanisms of recovery remain incompletely understood, and accurate prognostication continues to challenge clinicians.^1–3^ Contemporary neurocritical care strategies emphasize the mitigation of secondary neurologic injury through multimodal neurologic monitoring and serial neurologic examinations, including repeated assessment of the Glasgow Coma Scale (GCS) score.^4–6^ However, these tools provide only partial insight into injury severity and recovery potential, as children presenting with low initial GCS scores can, in some cases, go on to achieve unexpectedly favorable outcomes.^7,8^ Accordingly, there is a pressing need for objective, reproducible biomarkers that can more accurately characterize the severity of pediatric sTBI, monitor its dynamic course, and anticipate neurologic outcomes.^9,10^ To date, most biomarker investigations have centered on adult populations, evaluated a limited number of candidate proteins, and relied on single timepoints, leaving gaps in our understanding of the temporal evolution of circulating biomarkers in pediatric sTBI.

Emerging high-throughput proteomic technologies, including aptamer-based assays, provide a promising opportunity to address these gaps. These assays use short, single-stranded oligonucleotides that fold into defined three-dimensional conformations, enabling high-affinity and high-specificity binding to target proteins.^11^ Functioning analogously to antibodies but with lower cross-reactivity, aptamers allow quantification of thousands of proteins simultaneously from small plasma samples. Their high sensitivity for detecting low-abundance proteins enable more comprehensive quantification of the plasma proteome, helping overcome the throughput and scalability limitations of conventional methods such as enzyme-linked immunoassays (ELISA) and mass spectrometry.^11,12^ Multiplex proteomic approaches have already demonstrated potential in adult sTBI, identifying candidate biomarkers of neuroinflammation, hypoxia, and cellular injury – with inflammatory mediators such as complement factors emerging as prognostic indicators.^13,14^ Applying aptamer-based proteomic profiling to pediatric sTBI may enable more nuanced characterization of molecular injury phenotypes based on specific and measurable pathobiological mechanisms. This approach could facilitate biomarker discovery and ultimately support advances in precision neurocritical care.^15,16^

In this pilot study, we used aptamer-based proteomic profiling to characterize the plasma proteome in a convenience sample of children with sTBI at 24- and 72-hours following injury, compared with healthy controls. The primary objective of our pilot study was to determine the temporal evolution of the circulating proteome during the acute phase of injury. A secondary objective was to assess whether early proteomic signatures correlated with functional neurologic outcomes at hospital discharge and 3 months post-injury. We hypothesized that sTBI would induce the release of low-abundance, brain-derived proteins detectable using an aptamer-based platform, and that distinct proteomic profiles – and their evolution over time – would be associated with early clinical trajectories and short-term outcomes.

## Methods

Study approval was obtained from the Institutional Review Board (IRB) at Washington University in St. Louis (IRB# 201105185 and 201107146). Written informed consent was obtained from each participant’s parent or legal guardian prior to enrollment. Recruitment, data collection, and sample collection were all performed at Washington University in St. Louis. Following completion of these study activities, data were analyzed at Washington University in St. Louis and at Children’s Hospital Los Angeles (IRB # CHLA-23-00194). All study procedures adhered to the ethical standards of the responsible committee on human experimentation and were consistent with the Helsinki Declaration of 1975.^17^

### Study design and participants

This prospective, non-randomized, repeated-measures case-control pilot study included patients younger than 18 years admitted to the Washington University in St. Louis Pediatric Intensive Care Unit (PICU) between January 2014 and December 2019 with severe traumatic brain injury, defined by a post-resuscitation GCS score ≤8. Exclusion criteria comprised prior head trauma, pre-existing neurologic or severe chronic non-neurologic conditions, spinal cord injury, or inability to verify the timing of sTBI. The analytic cohort comprised a convenience sample of 24 sTBI patients selected solely on the basis of availability of archived plasma samples at the pre-specified post-injury timepoints. These samples were derived from patients enrolled between 2016 and 2019. Healthy control participants were children without known acute or chronic illness who were recruited from a pediatrician’s office during routine health maintenance visits.

### Data collection

For patients with sTBI, plasma samples were prospectively collected at 24- and 72-h post-injury. For healthy controls, plasma was collected at a single timepoint. Data abstracted from the electronic health record (Epic Systems Corporation, Verona, WI) included age, sex, race, mechanism of injury, post-resuscitation GCS, cardiac arrest within 72 h of injury, and Glasgow Outcome Scale – Extended, Pediatric Revision (GOS–E Peds) scores at hospital discharge and at 3 months follow-up. GCS scores were documented by the clinical care team and independently verified by the study investigators prior to enrollment. GOS–E Peds scores, which range from 1 to 8, with higher scores reflecting greater neurologic disability and poorer functional outcome, were assigned by trained research coordinators.^18^ Study investigators and research coordinators remained blinded to proteomic results throughout all outcome assessments. All data were de-identified before analysis.

### Plasma proteomic analysis

To minimize the impact of freeze-thaw cycles, primary plasma samples were aliquoted into smaller 0.5 mL volume and stored at −80 °C until a specific analysis was performed. Upon thawing, samples were analyzed using SomaScan Assay 1.3 K (SomaLogic Inc., Boulder, CO), an aptamer-based protein array that measures the relative concentrations of 1297 proteins.^12^ SomaScan output values represent relative fluorescence units reflecting normalized protein abundance rather than absolute protein concentrations. Samples were processed according to the SomaLogic Inc. recommended standard protocols for SomaScan Assay kits.^19^ Briefly, 50 µL of plasma from each patient was diluted with sample diluent and assay buffer into three separate wells of the assay plate at 40%, 1%, and 0.005%, targeting plasma proteins with variable abundance. Samples were then incubated with SOMAmer reagents. Non-specific binding was quenched, and the SOMAmer-protein complexes were hybridized to Agilent SureScan MicroArray slides, which were scanned at 5 µm resolution for Cyanine3 fluorescence. Gridding and image processing were performed using Agilent Feature Extraction software (version 10.7.3.1; Agilent Technologies, Santa Clara, CA). The results of SomaScan protein expression data were internally validated for precision and accuracy and are described elsewhere.^19^

### Data preprocessing

The raw signal values were normalized by the SomaLogic bioinformatics services team through the SomaScan standardization procedures, including hybridization normalization, plate calibration, median scaling, and SOMAmer calibration.^20,21^ Data quality was evaluated by examining signal variation across individual samples using hybridization probes, across multiple dilution bins, sample types, and plates relative to reference calibrators, and across individual SOMAmers compared with calibrator reference values within each plate. All investigators responsible for data collection and preprocessing were blinded to sample information.

### Statistical analysis

Descriptive statistics were used to summarize participant characteristics. Continuous variables were reported as medians with interquartile ranges and categorical variables were summarized as counts and percentages. Patient characteristics were compared using Student’s *t* tests for continuous variables and chi-square tests for categorical variables in GraphPad Prism (version 10.5.0, GraphPad Software, San Diego, CA). No formal imputation was performed for missing data given the exploratory pilot design. One-way or two-way analysis of variance (ANOVA) was used for comparisons involving more than two groups or variables, including analysis of normalized relative protein expression values and their derived principal component scores across demographic and clinical subgroups.

Standardized protein signal intensities were normalized using the quantile normalization procedure in R (version 4.3.2, R Foundation for Statistical Computing, Vienna, Austria) to harmonize distribution across samples and reduce inter-sample variability. To visualize differences among proteomic profiles of individual patients and assess group-level clustering, principal component analysis (PCA) was performed in Partek GS (version 7.0, Partek Inc., St. Louis, MO). PCA is a multivariate dimension-reduction technique that transforms correlated variables, in this case protein intensities, into a smaller set of uncorrelated variables known as principal components (PCs). Each PC is a weighted linear combination of the original variables, ordered by the proportion of total variance it explains. PC1 captures the greatest variance, PC2 captures the next greatest orthogonal variance, and so on, enabling projection of high-dimensional data into a lower-dimensional space while preserving dominant patterns. In this reduced space, each patient’s proteomic profile is represented as a single point, with the spatial relationships among points reflecting similarities or differences in overall protein expression patterns. F-values from ANOVA models comparing PC scores across clinical variables were used to assess the contribution of these factors to variation in PCA space.

To formally assess the degree of separation between patient groups, we calculated the Euclidean distance in the three-dimensional PCA-derived projection space using the centroid coordinates of the control group and the sTBI groups at 24- and 72-h post-injury. Euclidean distances measure the straight-line distance between points in multi-dimensional space, providing an interpretable metric of group dissimilarity. To assess the likelihood of observing the measured separation between serum proteomic profiles by chance, we performed a permutation test in which group labels were randomly reassigned 1000 times to generate a null distribution of Euclidean distances under the assumption of no true group separation. The observed distance was then compared to this null distribution, and a *p*-value was calculated as the proportion of permuted distances equal to or greater than the observed distance. We additionally reported the proportion of variance explained by each principal component to assess how well the dimensionality reduction captured relevant variability across samples.

Differentially expressed proteins (DEPs) across patient groups and timepoints were identified using the R package ‘limma’ with fitted and paired linear models.^22^ Comparison of protein expression values between patient groups were done through moderated *t*-tests with Bayes moderation to stabilize variance estimates, and paired *t*-tests were used for temporal comparisons. For comparisons involving more than two groups or multiple variables, one-way or two-way ANOVA was applied. The Benjamini and Hochberg false-discovery rate (FDR) procedure was used to adjust for multiple comparisons. Given the exploratory nature of this pilot study, a lenient threshold was applied to capture potentially relevant candidates for downstream validation, with significance defined as *p* < 0.05 and FDR-adjust *p*-value (*q*-value) <0.3. We also reported the number of proteins that remained significant under a more conservative FDR threshold of *q* < 0.05.

To explore the biological relevance of differentially expressed proteins, we first performed functional enrichment analyses on the sets of up- and downregulated proteins for each comparison. First, we used Metascape (https://metascape.org), a web-based gene annotation and pathway enrichment tool, to identify over-represented Gene Ontology (GO) terms and biological pathways.^23^ Metascape clusters similar terms based on shared gene membership and selects the most statistically significant term within each cluster to serve as a representative. This clustering approach facilitates high-level visualization of perturbed biological processes. Because several biological pathways contained both upregulated and downregulated proteins, they appeared enriched in both sets. We therefore examined the relative enrichment significance to determine whether specific pathways were predominantly activated or suppressed at each timepoint.

To complement the GO-based analysis and provide a broader interpretive framework, we next applied the COmprehensive Multi-omics Platform for Biological InterpretatiOn (CompBio; PercayAI Inc. v2.5, St. Louis, MO).^24^ CompBio uses a natural language processing algorithm to extract enriched biological themes from PubMed abstracts based on submitted protein lists. Unlike conventional enrichment tools limited to predefined ontologies, CompBio identifies statistically significant biological narratives through a literature-driven, probabilistic modeling approach. Enriched themes are grouped into higher-order clusters, offering contextual insight into key pathophysiologic processes associated with sTBI at 24- and 72-h post-injury timepoints. For each enriched theme, CompBio calculates a normalized enrichment score (NEScore) based on concept overlap, a Theme Score reflecting cumulative relevance and strength of supporting literature evidence, and an associated p-value. To better characterize neurologic injury biology, we focused interpretation on “brain-specific” clusters, which highlight neurobiological processes such as axon guidance, neuroinflammation, and synaptic signaling – features that may not be captured by GO-based tools alone.

Finally, to identify candidate biomarkers associated with neurologic outcome, we performed correlation analyses between protein abundance levels and GOS–E Peds scores at hospital discharge and at 3 months follow-up. Protein levels at 24- and 72-h post-injury were treated as independent variables, and GOS–E Peds scores were treated as continuous outcomes. Pearson correlation coefficients were calculated for each protein, and proteins associated with GOS–E Peds at a significance threshold of *p* < 0.001 were retained for model development.

To construct multivariable predictive models, we selected the top 30 proteins from each timepoint based on their univariate Pearson correlation rankings. Variable selection was performed using random forward selection via the ‘leaps’ R package. Final models were derived using ‘bestglm’, which implements best-subsets linear regression with internal cross-validation to mitigate overfitting. Model performance was evaluated by coefficient of determination (R²), cross-validated predictive power (q²), root mean square error (RMSE) and mean absolute error (MAE). Separate models were developed to predict GOS–E Peds scores at discharge and at 3 months follow-up, using proteomic data from either the 24- or 72-h timepoint. All analyses were performed in R (version 4.3.2), and visualizations were generated using ‘ggplot2’.

## Results

### Patient characteristics

**Fig. 1:**
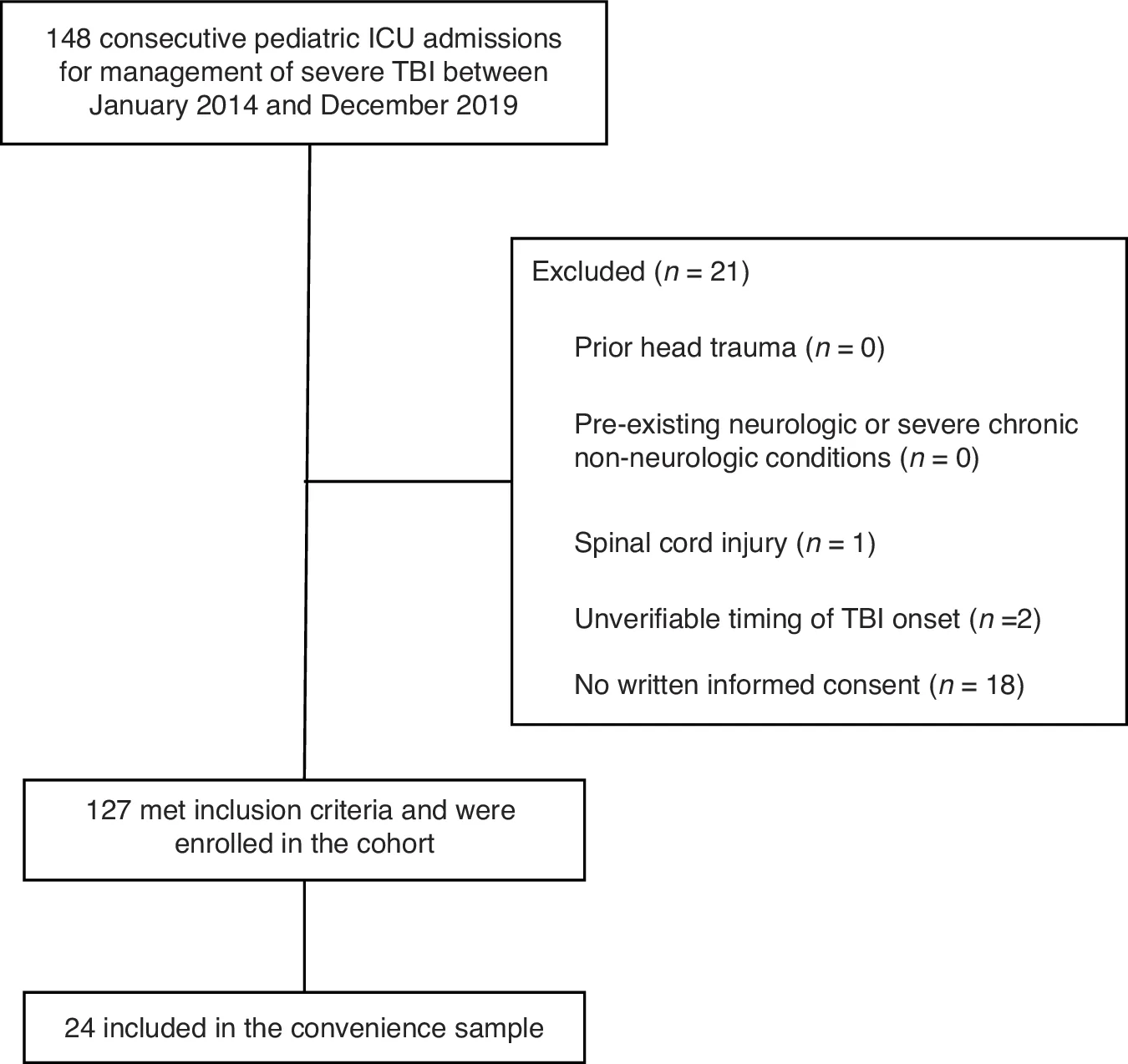
Flowchart detailing the inclusion of patients admitted to the intensive care unit for management of severe traumatic brain injury during the study period. ICU intensive care unit, TBI traumatic brain injury.

A total of 148 children were identified as having severe traumatic brain injury during the study period, of whom 127 met inclusion criteria and were enrolled in the study. Samples from a convenience sample of 24 sTBI patients were analyzed for this study. Samples from 4 control patients were also analyzed (Fig. 1). The median age of the sTBI cohort was 13.1 years (IQR 6.3, 16.8). The majority (*n* = 16, 66.7%) sustained injury via motor vehicle collisions, consistent with contemporary sTBI cohorts.^25^ Twenty of twenty-four (83.3%) patients underwent intracranial pressure (ICP) monitoring and ICP-directed therapies per institutional guidelines. All patients survived, but 3 patients who survived to hospital discharge were lost to follow-up at 3 months. Demographic and clinical characteristics are summarized in Table 1.

**Table 1:** Demographic, clinical, and outcome characteristics of children with severe traumatic brain injury

Patient characteristics	Severe TBI cohort (*n* = 24)
Demographics	
Age, years	13.4 (6.4, 16.8)
Female sex	11 (45.8)
Non-white race	8 (33.3)
Etiology of TBI	
Motor vehicle accident	16 (66.7)
Pedestrian accident	4 (16.7)
Gunshot wound	1 (4.2)
Fall	3 (12.5)
Clinical characteristics	
Non-accidental injury	1 (4.2)
Post-resuscitation GCS score	4.5 (3.0, 6.3)
CA within 72 h of TBI	1 (4.2)
GOS-E peds outcome scores	
Hospital discharge^a^	4.0 (3.0, 4.0)
3 months^a^	5.0 (3.0, 6.5)

Data are presented as *n* (%; valid percentage excluding missing values) and median (interquartile range).

*CA* cardiac arrest, *GCS* Glasgow coma scale, *GOS-E* Peds Glasgow outcome scale – extended, pediatric revision, *TBI* traumatic brain injury.

^a^GOS-E Peds Outcome Scores at hospital discharge and 3 months were missing for one and four patients, respectively.

### Plasma proteomic profiling of pediatric sTBI patients and controls

Principal component analysis of normalized plasma protein expression revealed modest separation between groups (Supplementary Fig. S1).^26^ While sTBI and control samples did not form completely distinct clusters, 95% confidence ellipsoids demonstrated progressive divergence between the 24- and 72-h timepoints. Within-subject trajectories between 24- and 72-h showed consistent directional shifts, supporting a temporally dynamic proteomic response to injury.

The first three principal components accounted for 34.6% of the total variance in plasma protein expression, with the greatest separation observed along PC1 and PC2 (Supplementary Table S1A–C). Quantitative comparisons of group centroids showed Euclidean distances of 14 units between the 24 h post-injury group and healthy controls, 24 units between the 72 h post-injury group and controls, and 14 units between the 24- and 72-h post-injury groups. Among demographic and clinical variables, post-injury timepoint accounted for the greatest proportion of variance (*F* = 4.6), followed by patient age (*F *= 3.3). Race, sex, mechanism of injury, and post-resuscitation GCS score contributed comparatively less (Supplementary Fig. S2).

### Age-associated proteomic changes

To evaluate the influence of age, we examined correlations between patient age and plasma protein abundance at 24- and 72-h post-injury. At 24 h, 51 proteins were significantly associated with age (*r* = −0.80 to 0.84; Supplementary Table S2A). Positively correlated proteins included soluble tyrosine-protein kinase receptor Tie-1 (TIE1, *r* = 0.84), a vascular endothelial receptor, ciliary neurotrophic factor receptor subunit alpha (CNTFRα, *r* = 0.83), which mediates neurotrophic signaling, and chordin-like protein 1 (CHRDL1, *r* = 0.81), an astrocyte-derived modulator of synaptic maturation. Negatively correlated proteins included C-C motif chemokine 22 (CCL22, *r* = −0.80), a chemokine involved in immune trafficking, serine protease 27 (PRSS27, *r* = −0.78), a protease linked to extracellular matrix turnover, and high affinity nerve growth factor receptor (NTRK1, *r* = −0.78), a neuronal growth factor critical for neuronal survival.

At 72 h, 32 proteins were significantly age-associated (*r* = −0.84 to 0.92; Supplementary Table S2B). Stromal cell-derived factor 1 (CXCL12, *r* = 0.92), a chemokine that guides neuronal and vascular development, remained strongly correlated, along with CHRDL1 (*r* = 0.89) and cystatin-SA (CST2, *r* = 0.83), a protease inhibitor with putative roles in tissue remodeling. Negatively correlated proteins included casein kinase II subunit alpha (CSNK2A1, *r* = −0.84), a kinase regulating synaptic and transcriptional processes, alpha-2-macroglobulin receptor-associated protein (LRPAP, *r* = −0.76), which modulates receptor-mediated clearance at the blood-brain barrier, and interleukin-17D (IL-17D, *r* = −0.75), a cytokine implicated in neuroinflammation. Together, these data indicated that patient age correlates with proteomic profiles across multiple brain-relevant pathways in pediatric sTBI.

### Differentially expressed proteins across timepoints

**Fig. 2:**
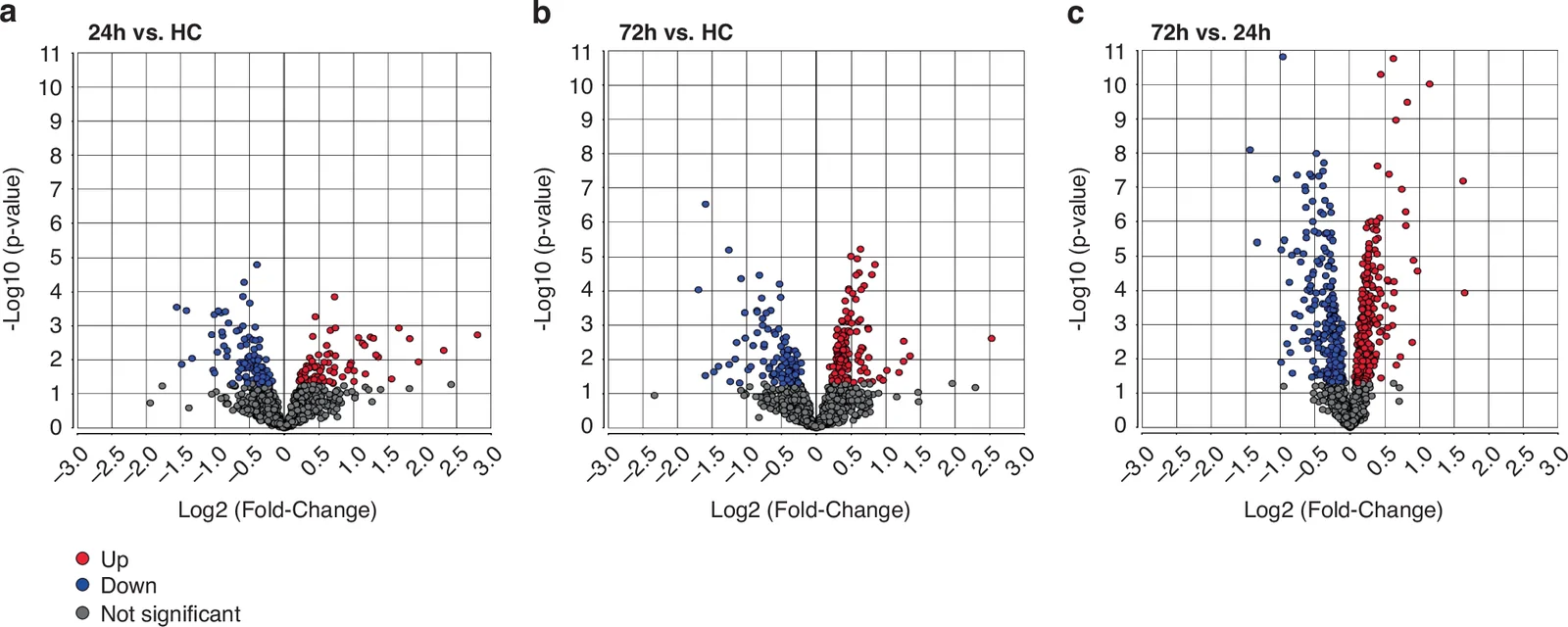
Volcano plots showing the magnitude of plasma protein change between severe traumatic brain injury patients at 24- and 72-h post-injury versus healthy controls, and between the two timepoints. Upregulated (red) and downregulated (blue) proteins were identified based on significant abundance changes at *p* < 0.05.

Differential expression analysis identified 634 unique proteins with significant changes across three primary comparisons: sTBI vs. control at 24 h, sTBI vs. control at 72 h, and 72- vs. 24-h within the sTBI group (Fig. 2). At 24 h, 161 proteins were differentially expressed compared to controls (70 upregulated, 91 downregulated). Using the exploratory threshold defined a priori (*p* < 0.05, *q* < 0.3), 138 proteins met a fold-change (FC) ≥ 1.25, of which 64 exceeded ≥1.5. Under a more conservative threshold (*q* < 0.05), only 4 proteins remained significant at ≥1.25 and 2 at ≥1.5. At 72 h, 231 proteins were differentially expressed (136 upregulated, 95 downregulated); 184 met the ≥1.25 FC criterion and 72 met ≥1.5 under exploratory criteria, while 51 and 28 proteins, respectively, remained significant at the conservative threshold.

Paired within-subject analysis between 72- and 24-h post-injury revealed 548 DEPs (329 upregulated, 219 downregulated), with 440 remaining significant after FDR correction. Only 35 proteins were shared across all three comparisons (Supplementary Fig. S3A), underscoring the temporal specificity of the plasma proteomic response. Heat map visualization highlighted diverse trajectories of early, late, and sustained proteomic dysregulation (Supplementary Fig. S3B).

**Fig. 3:**
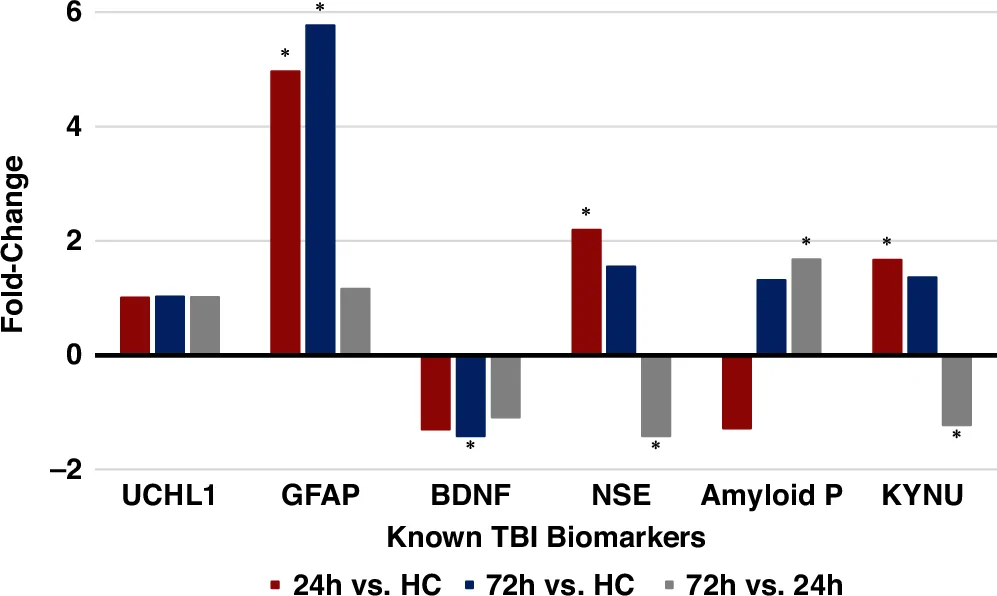
Temporal changes in established traumatic brain injury biomarkers. Fold-change values for selected established TBI biomarkers comparing 24 h vs. healthy controls (blue), 72 h vs. healthy controls (gray), and 72 h vs. 24 h (black) following pediatric severe TBI.

Several established TBI biomarkers demonstrated distinct temporal patterns following injury (Fig. 3, Supplementary Table S3). Glial fibrillary acidic protein (GFAP) was markedly elevated at both 24- and 72-h, consistent with astroglial damage.^9^ Neuronal injury was reflected by increased neuron-specific enolase (NSE) at 24 h, which declined by 72 h.^9^ The metabolic enzyme kynureninase (KYNU), involved in clearance of neuroexcitotoxic metabolites, followed a similar early peak and subsequent decline.^27,28^ In contrast, brain-derived neurotrophic factor (BDNF) was reduced at both timepoints, suggesting persistent suppression of pathways supporting neuronal survival and synaptic plasticity.^9^

### Biological theme enrichment

**Fig. 4:**
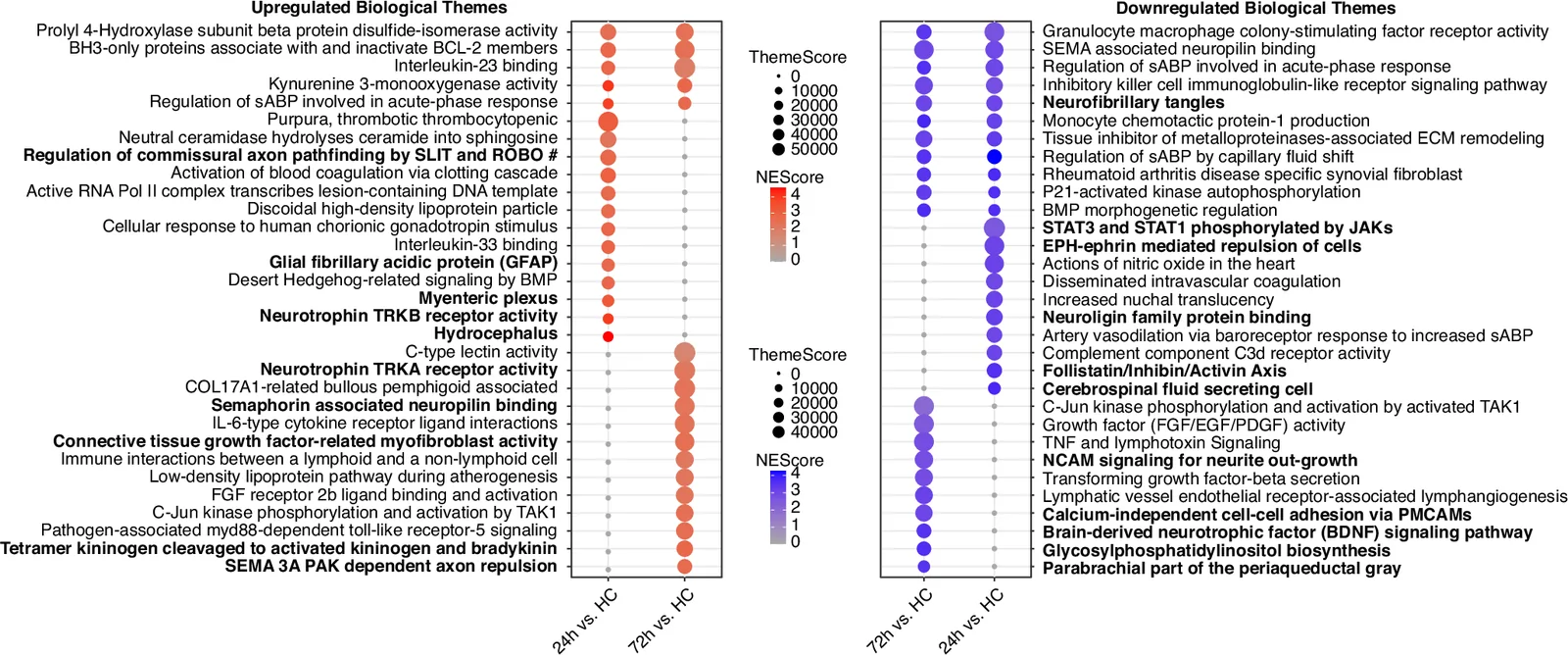
Enriched biological themes following pediatric sTBI. Upregulated (red) and downregulated (blue) pathways at 24 h and 72 h vs. healthy controls.

At 24 h post-injury, key brain-specific biological pathways were differentially regulated (Fig. 4). “Glial Fibrillary Acidic Protein” signaling was significantly upregulated (NEScore 2.68, Theme Score 13,149, *p* = 0.011), consistent with astrocytic activation, as were “Hydrocephalus-Related Signaling” (NEScore 4.49, Theme Score 5971, *p* = 0.012), indicating early disturbances in cerebrospinal fluid (CSF) dynamics, and “Kynurenine 3-Monooxygenase Activity” (NEScore 3.20, Theme Score 11,949, *p* = 0.003), reflecting increased production of neurotoxic metabolites along the kynurenine pathway.^27^ In contrast, “Neurofibrillary Tangle-Associated Signaling” (NEScore 3.00, Theme Score 21,376, *p* < 0.001) and “CSF-Secreting Cell Signaling” (NEScore 2.49, Theme Score 15,944, *p* = 0.003) were significantly downregulated, reflecting suppression of pathways involved in cytoskeletal integrity and CSF production, respectively. Systemically, “Purpura, Thrombotic Thrombocytopenic”-related themes were significantly upregulated (NEScore 3.07, Theme Score 44,231, *p* = 0.001), while “Disseminated Intravascular Coagulation” (DIC) pathways were downregulated (NEScore 2.59, Theme Score 35,953, *p* = 0.002); together, these findings point to early endothelial dysfunction and dysregulation of coagulation control mechanisms.

By 72 h post-injury, the proteomic profile shifted to reflect evolving neurobiological responses (Fig. 4). “Neurotrophin Tropomyosin Receptor Kinase A (TRKA) Receptor Activity” was significantly upregulated (NEScore 2.14, Theme Score 50,045 *p* < 0.001), consistent with re-engagement of neurotrophic signaling. However, several brain-specific pathways remained significantly downregulated, including “Brain-Derived Neurotrophic Factor” signaling (NEScore 3.46, Theme Score 16,584, *p* < 0.001), “Neural Cell Adhesion Molecule (NCAM) Signaling For Neurite Outgrowth” (NEScore 2.76, Theme Score 31,436, *p* < 0.001), and “Neurofibrillary Tangle”-associated signaling (NEScore 3.01, Theme Score 21,443, *p* < 0.001), indicating persistent suppression of signaling related to neuronal survival, synaptic organization, and cytoskeletal structure in the subacute phase of pediatric sTBI.

### Protein biomarkers correlated with injury severity and outcomes

**Fig. 5:**
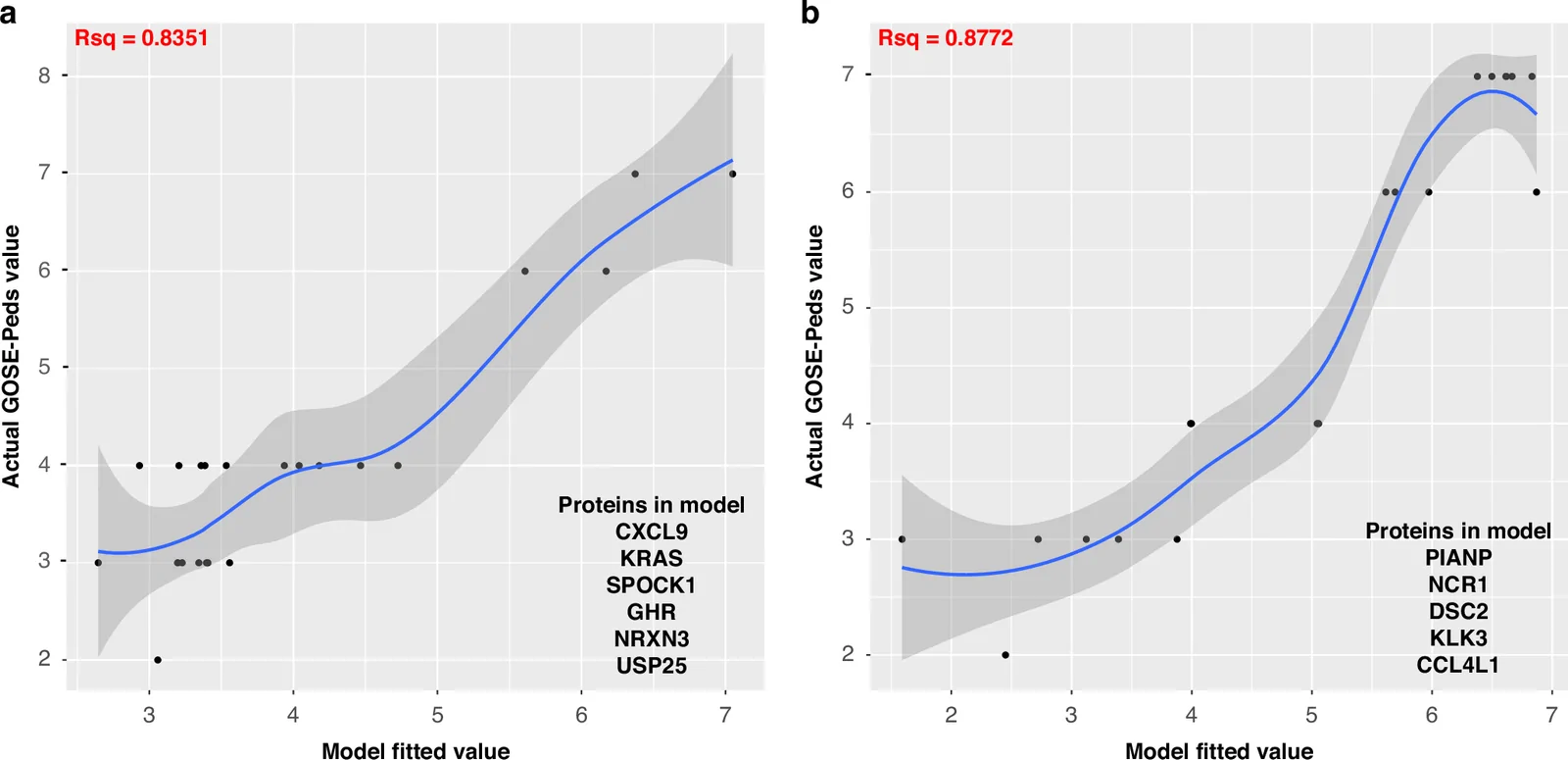
Regression models predicting GOS–E Peds scores using plasma proteomic profiles at 72 h for discharge and 24 h for 3-month outcomes in pediatric severe traumatic brain injury patients. **a** 72 h for discharge; **b** 24 h for 3-month outcome. CCL4L1 C-C motif chemokine ligand 4-like 1, CXCL9 C-X-C motif chemokine ligand 9, DSC2 desmocollin-2, GHR growth hormone receptor, GOSE Glasgow outcome scale – extended, pediatric revision, KLK3 kallikrein-related peptidase 3, KRAS Kirsten rat sarcoma viral oncogene homolog, NCR1 natural cytotoxicity triggering receptor 1, NRXN3 neurexin-3, PIANP PILR alpha-associated neural protein, Rsq square of the Pearson correlation coefficient, SPOCK1 testican-1, USP25 ubiquitin-specific peptidase 25.

Among survivors of sTBI, multivariable regression modeling identified distinct protein panels associated with GOS–E Peds scores. A model incorporating protein measurements obtained at 72 h post-injury predicted neurologic outcome at hospital discharge with high accuracy (R² = 0.8351; Fig. 5a). The six most informative proteins in this model were testican-1 (SPOCK1), neurexin-3 (NRXN3), Kirsten rat sarcoma viral oncogene homolog (KRAS), ubiquitin-specific peptidase 25 (USP25), growth hormone receptor (GHR), and C-X-C motif chemokine ligand 9 (CXCL9). Among these, SPOCK1 corresponded to the enriched pathway “Connective Tissue Growth Factor-Related Myofibroblast Activation”, and NRXN3 aligned with the downregulated theme “NCAM Signaling for Neurite Outgrowth”.

A separate regression model incorporating 24-h post-injury plasma levels of 5 proteins predicted GOS–E Peds scores at 3 month follow-up with similarly strong performance (R² = 0.8772; Fig. 5b). This model included PILR alpha-associated neural protein (PIANP), natural cytotoxicity triggering receptor 1 (NCR1), desmocollin-2 (DSC2), kallikrein-related peptidase 3 (KLK3), and C-C motif chemokine ligand 4-like 1 (CCL4L1). CCL4L1 was associated with the theme “CCL18/Anti-Inflammatory TRKB Receptor Signaling”, KLK3 with “Tetramer Kininogen Cleavage to Activated Kininogen and Bradykinin”, and DSC2 with “Calcium-Independent Cell-Cell Adhesion Via Plasma Membrane Cell Adhesion Molecules (PMCAMS)”. Several of these proteins were also differentially expressed at their respective timepoints, reinforcing their mechanistic relevance in the context of pediatric sTBI.

## Discussion

In this prospective, non-randomized, repeated-measures case-control pilot study, we applied aptamer-based proteomic profiling to characterize circulating plasma proteins in children with sTBI at two clinically relevant timepoints: 24- and 72-h post-injury. When comparing these two timepoints, we observed dynamic and time-dependent shifts in protein expression, revealing evolving biologic responses not captured by traditional metrics.

PCA demonstrated progressive divergence between the proteomic profiles of sTBI patients and controls, with the greatest separation observed at 72 h post-injury. Among demographic and clinical variables, post-resuscitation GCS scores contributed minimally to proteomic variance – likely reflecting cohort homogeneity, as all patients presented with coma (GCS score ≤8). This finding reinforces previous reports that GCS offers limited prognostic granularity in pediatric sTBI and aligns with recent recommendations from the National Institute of Child Health and Human Development and National Institute for Neurologic Disorders and Stroke working group to move beyond simplistic GCS-based severity categories in favor of more nuanced classifications incorporating blood-based biomarkers.^29–31^ In contrast, patient age accounted for a substantial proportion of proteomic variance, with 88 proteins significantly correlated with age across both timepoints. Prior experimental and clinical studies have demonstrated that brain maturation is associated with differences in cerebral metabolism and physiological responses to biomechanical injury, as well as with age-dependent variation in the concentration and temporal kinetics of established brain injury biomarkers, including NSE, S100B, and Ubiquitin C-terminal Hydrolase L1 (UCH-L1).^32–36^ Additional evidence indicates that the immature brain exhibits distinct neuroinflammatory responses to injury, characterized by developmental differences in cytokine signaling, glial activation, and susceptibility to secondary insults.^8,37^ Collectively, these findings highlight the importance of age-stratified reference frameworks to distinguish normative developmental variation from injury-associated proteomic changes.^38^

Differential expression analysis identified 634 unique proteins with significant changes across injury-control and within-subject comparisons, including well-characterized biomarkers of neurologic injury such as GFAP, NSE, and BDNF. Importantly, our analysis also revealed multiple novel candidate biomarkers and associated biological themes not previously described in pediatric TBI. These findings support the biological validity of the aptamer platform for detecting both clinically relevant and exploratory brain-related pathology from peripheral plasma, an especially valuable feature in pediatric populations where CSF access is limited and sample volumes are small.^9,39^ At 24 h post-injury, brain-specific proteomic alterations reflected acute astrocytic activation (i.e., GFAP), impaired CSF dynamics, and suppression of cytoskeletal organization (i.e., neurofibrillary tangles), consistent with early structural and functional disruption. Concurrent systemic signatures indicated endothelial dysfunction and dysregulation of coagulation, both of which are clinically relevant in the context of pediatric TBI where bleeding and secondary ischemia are major concerns. By 72 h, we observed a modest shift toward reparative signaling, with increased representation of neurotrophic and structural pathways (i.e., BDNF, NCAM). However, persistent downregulation of key brain-specific processes involved in synaptic organization and neuronal survival suggests that regenerative mechanisms remain only partially engaged during the subacute phase.

The temporally distinct shifts in brain and systemic proteomic signaling observed in this study suggest that pediatric sTBI follows heterogeneous trajectories of inflammation and repair, rather than a uniform course of recovery. These dynamic molecular responses may reflect distinct endotypes of injury–paralleling observations in adult TBI, pediatric sepsis, and acute respiratory distress syndrome, where patients with similar clinical presentations demonstrate divergent biological pathways that influence outcomes.^40–44^ In our pilot cohort, time-resolved proteomic profiling identified multi-protein signatures at 24- and 72-h post-injury that were associated with neurologic outcomes at 3 month follow-up and hospital discharge, respectively. These findings raise the possibility that molecular phenotyping, particularly when captured across time, may expose clinically silent but biologically meaningful patterns of injury response that are not evident through conventional monitoring.^8^ Rather than relying on single analytes, composite biomarker panels incorporating temporal data may provide greater prognostic precision and better support future efforts in risk stratification and development of targeted therapies aimed at minimizing secondary neurologic injury.^38,45,46^

This study has several important limitations. First, while proteomic data offer valuable biological insight, they do not establish causality. Protein expression is subject to both technical variability and physiological complexity, and group-level differences should be interpreted with caution. To reduce the risk of overinterpretation, we emphasized enrichment of biological pathways rather than individual analytes. Second, the absence of a critically ill trauma control group without brain injury limits our ability to distinguish TBI-specific proteomic alterations from non-specific systemic responses to trauma. Third, the small sample size and single-center design limit statistical power and generalizability. In addition, this pilot study was not powered to evaluate the contribution of specific injury mechanisms, such as abusive head trauma or diffuse hypoxic ischemic brain injury following cardiac arrest, to the observed variability in proteomic profiles. These important injury characteristics should be examined in future studies with larger and more diverse cohorts. Fourth, the analysis did not incorporate neuroimaging features at the time of sampling, including established computed tomography-based injury severity classifications such as the Marshall or Rotterdam scores. Given the relevance of structural injury burden in sTBI, future investigations should incorporate standardized neuroimaging metrics alongside biomarker data to enable more comprehensive phenotyping, improve biological interpretability, and better link molecular signatures to underlying injury patterns and clinical outcomes. Finally, the analysis was limited to two sampling timepoints – 24- and 72-h post-injury – which may not fully capture the temporal dynamics of injury evolution. In addition, outcomes were assessed only at discharge and 3 months follow-up; longer-term follow-up will be necessary to determine the sustained prognostic utility of early plasma proteomic signatures.

## Conclusion

This pilot study demonstrates the feasibility and biological validity of aptamer-based plasma proteomics to characterize evolving molecular responses following pediatric sTBI. We identified distinct temporal shifts in both brain-specific and systemic signaling pathways within the first 72 h post-injury, with patient age contributing to proteomic variability across timepoints. These findings provide proof-of-concept that high-dimensional, temporally resolved proteomic profiling can capture clinically meaningful signatures of injury biology in pediatric sTBI. Our results support the utility of applying aptamer-based technology to future efforts in biomarker discovery, risk stratification, and the development of precision medicine approaches in sTBI and pediatric neurocritical care.

## Supplementary information

Supplementary Information

Supplementary Information

## Data Availability

The datasets generated during and/or analyzed during the current study are available from the corresponding author on reasonable request.
