# Supplementary material for: Aptamer-based proteomics in pediatric patients with severe traumatic brain injury: a pilot study
Source: Pediatr Res. 2026 Feb 19;100(3):1243–51. doi: 10.1038/s41390-026-04798-8 (PMC13672064; doi:10.1038/s41390-026-04798-8)
Supplement: Supplementary file 1 [file 41390_2026_4798_MOESM1_ESM.pdf]

TABLE OF CONTENTS

Figure S1. Three-dimensional principal component analysis of plasma proteomic profiles. 2

Figure S2. Demographic factors contributing to plasma proteomic variability in children withsevere traumatic brain injury. 3

Figure S3. Temporal specificity and dynamic trajectories of differentially expressed plasma proteins following pediatric severe traumatic brain injury. 4

Table S1A. Proteins with the highest PC1 loadings from principal component analysis of plasma protein expression following pediatric severe traumatic brain injury. 5

Table S1B. Proteins with the highest PC2 loadings from principal component analysis of plasma protein expression following pediatric severe traumatic brain injury. 7

Table S1C. Proteins with the highest PC3 loadings from principal component analysis of plasma protein expression following pediatric severe traumatic brain injury. 9

Table S2A. Differentially expressed proteins significantly correlated with age in covariate-adjusted analysis at 24 hours post-injury. Bolded proteins are statistically significant at $p < 0.05$ and $q < 0.3$. 11

Table S2B. Differentially expressed proteins significantly correlated with age in covariate-adjusted analysis at 72 hours post-injury. Bolded proteins are statistically significant at $p < 0.05$ and $q < 0.3$. 19

Table S3. Fold-change and statistical significance of established traumatic brain injury biomarkers at 24- and 72-hours post-injury compared with healthy controls and between timepoints. Bolded values are statistically significant at $p < 0.05$. 25

Figure S1. Three-dimensional principal component analysis of plasma proteomic profiles. PCA plot showing clustering of pediatric severe TBI patients at 24- (blue) and 72-hours (red) post-injury versus healthy controls (gray).

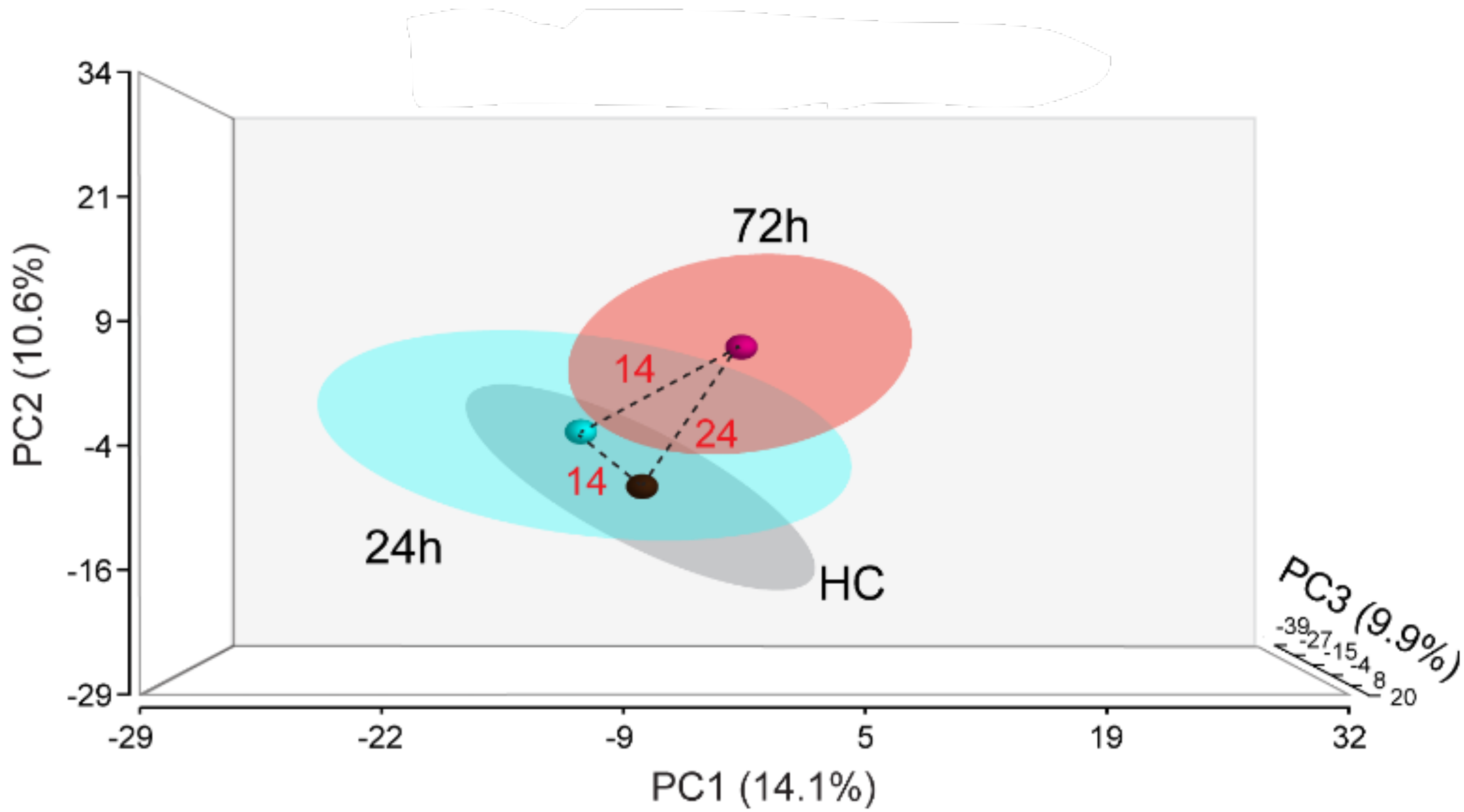

HC = healthy controls; PC = principal component; 24h = 24 hours; 72h = 72 hours

Dashed lines show Euclidean distances between group centroids; ellipsoids represent one standard deviation. Percent variance explained is shown on each axis.

Figure S2. Demographic factors contributing to plasma proteomic variability in children with severe traumatic brain injury.

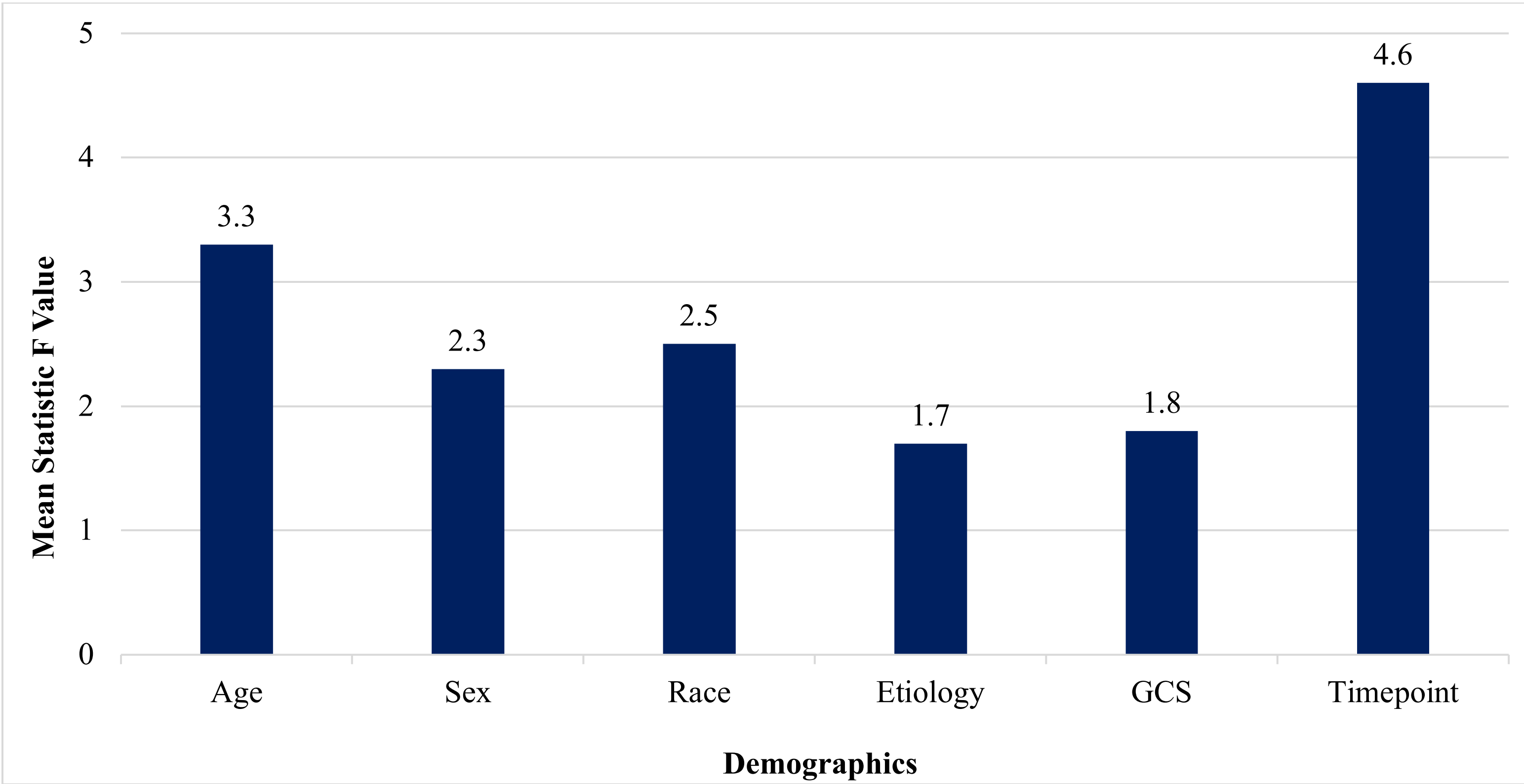

GCS = Glasgow coma scale

Figure S3. Temporal specificity and dynamic trajectories of differentially expressed plasma proteins following pediatric severe traumatic brain injury. (A) Venn diagram illustrating overlaps in differentially expressed plasma proteins between pediatric severe TBI patients and healthy controls at 24 hours and 72 hours post-injury, as well as between time points. (B) comparative heat maps of 634 differentially expressed proteins demonstrating distinct patterns of early (24h), late (72h), and sustained (24-72h) proteomic alterations.

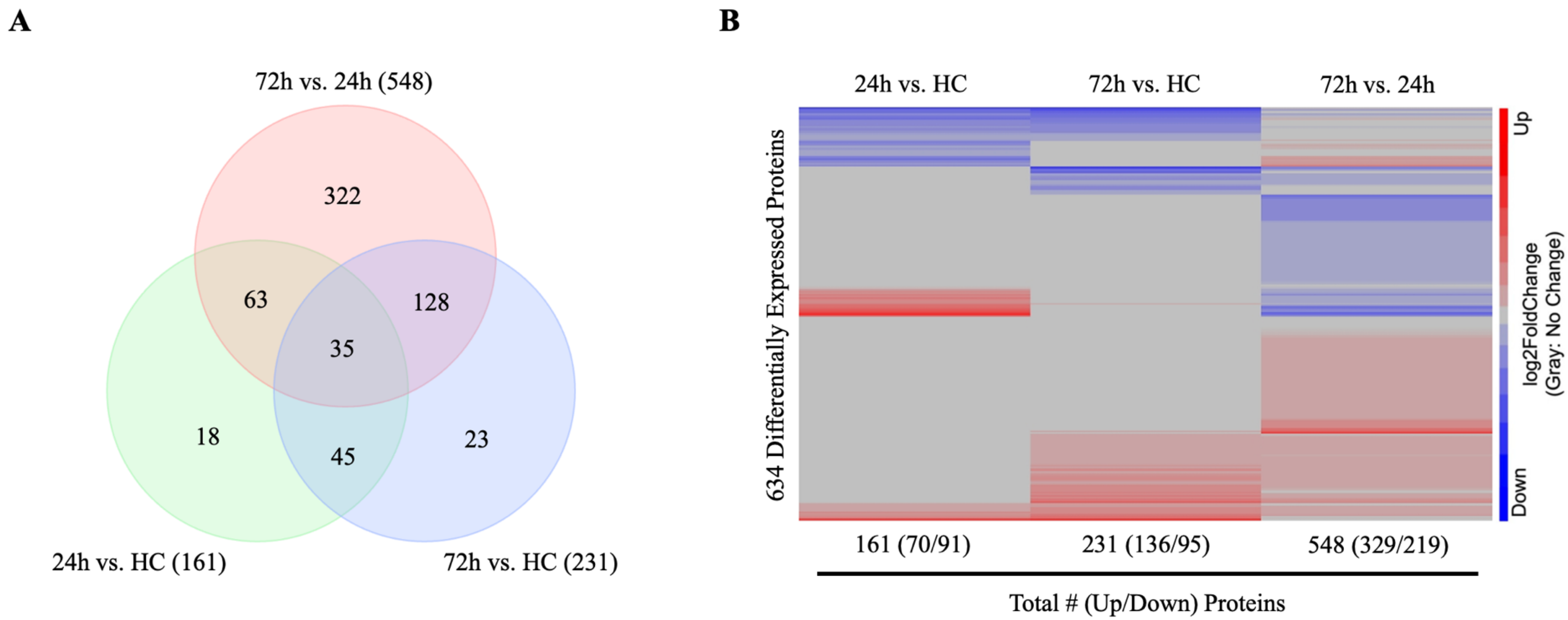

Proteins without significant fold-change differences are shaded in **gray**. **Red** = increased relative abundance; **blue** = decreased relative abundance. Numbers below each comparison denote the total number of differentially expressed proteins, with the numbers of upregulated and downregulated proteins shown in parentheses.

Abbreviations as in Figure S1.

Table S1A. Proteins with the highest PC1 loadings from principal component analysis of plasma protein expression following pediatric severe traumatic brain injury.

Aptamer Sequence ID	Entrez Gene Symbol	Target Full Name	PC1 Loading Values
seq.3363.31	CSK	Tyrosine-protein kinase CSK	0.873996
seq.10364.6	SMAD2	Mothers against decapentaplegic homolog 2	0.862115
seq.3416.2	BTK	Tyrosine-protein kinase BTK	0.836451
seq.4294.16	SPHK1	Sphingosine kinase 1	0.836348
seq.5013.2	CLIC1	Chloride intracellular channel protein 1	0.832067
seq.3866.7	SBDS	Ribosome maturation protein SBDS	0.829685
seq.2855.49	MAPK3	Mitogen-activated protein kinase 3	0.801516
seq.5245.40	PRKAA2 PRKAB2 PRKAG1	AMP Kinase (alpha2beta2gamma1)	0.796831
seq.4220.39	FER	Tyrosine-protein kinase Fer	0.78731
seq.3844.2	PPIA	Peptidyl-prolyl cis-trans isomerase A	0.777926
seq.3903.49	SNX4	Sorting nexin-4	0.772039
seq.3210.1	METAP1	Methionine aminopeptidase 1	0.771879
seq.3420.21	CA13	Carbonic anhydrase 13	0.771633
seq.4209.60	VTA1	Vacuolar protein sorting-associated protein VTA1 homolog	0.768718
seq.3469.74	RPS6KA3	Ribosomal protein S6 kinase alpha-3	0.760176
seq.3453.87	LYN	Tyrosine-protein kinase Lyn	0.759578
seq.5885.55	EIF4H	Eukaryotic translation initiation factor 4H	0.759514
seq.3872.2	TPT1	Translationally-controlled tumor protein	0.757446
seq.4460.8	PDPK1	3-phosphoinositide-dependent protein kinase 1	0.751888
seq.7660.21	TPM4	Tropomyosin alpha-4 chain	0.74952
seq.3480.7	DUSP3	Dual specificity protein phosphatase 3	0.746492
seq.3381.24	LYN	Tyrosine-protein kinase Lyn, isoform B	0.746448
seq.3848.14	GAPDH	Glyceraldehyde-3-phosphate dehydrogenase	0.746104

seq.5488.74	SRC	Proto-oncogene tyrosine-protein kinase Src	0.744041
seq.4188.1	AKR7A2	Aflatoxin B1 aldehyde reductase member 2	0.742089
seq.2870.29	RAC1	Ras-related C3 botulinum toxin substrate 1	0.735631
seq.3379.29	PRKCI	Protein kinase C iota type	0.728602
seq.5275.28	VAV1	Proto-oncogene vav	0.716123
seq.4240.31	PKM2	Pyruvate kinase PKM	0.715171
seq.3593.72	CASP3	Caspase-3	0.708888
seq.5464.52	GRB2	Growth factor receptor-bound protein 2	0.708045
seq.9169.14	SUMO3	Small ubiquitin-related modifier 3	0.699151
seq.3441.64	GSK3A GSK3B	Glycogen synthase kinase-3 alpha/beta	0.680278
seq.3115.64	MAPK1	Mitogen-activated protein kinase 1	0.667867
seq.3836.51	UFM1	Ubiquitin-fold modifier 1	0.665989
seq.5256.86	PDE5A	cGMP-specific 3',5'-cyclic phosphodiesterase	0.665754
seq.4963.19	ARPP19	cAMP-regulated phosphoprotein 19	0.665706
seq.5475.10	PRKCB	Protein kinase C beta type (splice variant beta-II)	0.664693
seq.3868.8	SGTA	Small glutamine-rich tetratricopeptide repeat-containing protein alpha	0.664212
seq.4292.5	NAPA	Alpha-soluble NSF attachment protein	0.660231
seq.4258.15	PA2G4	Proliferation-associated protein 2G4	0.660188

Table S1B. Proteins with the highest PC2 loadings from principal component analysis of plasma protein expression following pediatric severe traumatic brain injury.

Aptamer Sequence ID	Entrez Gene Symbol	Target Full Name	PC2 Loading Values
seq.2728.62	LAMA1 LAMB1 LAMC1	Laminin	0.770973
seq.14131.37	EFNB2	Ephrin-B2	0.761104
seq.3795.6	ADAM9	Disintegrin and metalloproteinase domain-containing protein 9	0.755697
seq.5440.26	TNNI2	Troponin I, fast skeletal muscle	0.740349
seq.3004.67	PDCD1LG2	Programmed cell death 1 ligand 2	0.720288
seq.2615.60	EFNA5	Ephrin-A5	0.714321
seq.3405.6	UFC1	Ubiquitin-fold modifier-conjugating enzyme 1	0.698201
seq.3799.11	CA3	Carbonic anhydrase 3	0.692846
seq.4962.52	CDNF	Cerebral dopamine neurotrophic factor	0.691863
seq.8465.52	CTSH	Cathepsin H	0.684549
seq.5121.3	SEMA6B	Semaphorin-6B	0.682273
seq.2816.50	BCAM	Basal Cell Adhesion Molecule	0.676979
seq.3042.7	MB	Myoglobin	0.676595
seq.2744.57	IGHG1 IGHG2 IGHG3 IGHG4 IGK2 IGL2	Immunoglobulin G	0.672639
seq.5122.92	SEMA6A	Semaphorin-6A	0.670635
seq.3074.6	LBP	Lipopolysaccharide-binding protein	0.670304
seq.3073.51	IL18BP	Interleukin-18-binding protein	0.670157
seq.3059.50	TNFSF13B	Tumor necrosis factor ligand superfamily member 13B	0.667173
seq.4908.6	ENG	Endoglin	0.663108
seq.2670.67	CKM	Creatine kinase M-type	0.659912
seq.3028.36	CCL23	Ck-beta-8-1	0.656282

seq.5864.10	ALDOA	Fructose-bisphosphate aldolase A	0.654947
seq.3324.51	LY9	T-lymphocyte surface antigen Ly-9	0.65367
seq.5115.31	RELT	Tumor necrosis factor receptor superfamily member 19L	0.648582
seq.4976.57	CRK	Adapter molecule crk	0.642724
seq.3714.49	CKB CKM	Creatine kinase M-type:Creatine kinase B-type heterodimer	0.637154
seq.4559.64	KYNU	Kynureninase	0.620663
seq.2972.57	BMP7	Bone morphogenetic protein 7	0.619146
seq.4232.19	IGF1R	Insulin-like growth factor 1 receptor	0.618123
seq.14157.21	YWHAE	14-3-3 protein epsilon	0.61716
seq.2828.82	SPINT1	Kunitz-type protease inhibitor 1	0.612986
seq.3050.7	VWF	von Willebrand factor	0.612954
seq.9171.11	CSRP3	Cysteine and glycine-rich protein 3	0.612512
seq.4834.61	EPHA2	Ephrin type-A receptor 2	0.610084
seq.4324.33	CST2	Cystatin-SA	0.609615
seq.3853.56	MDH1	Malate dehydrogenase, cytoplasmic	0.604484
seq.13122.19	FLRT2	Leucine-rich repeat transmembrane protein FLRT2	0.600737

Table S1C. Proteins with the highest PC3 loadings from principal component analysis of plasma protein expression following pediatric severe traumatic brain injury.

Aptamer Sequence ID	Entrez Gene Symbol	Target Full Name	PC3 Loading Values
seq.3859.50	PSMA1	Proteasome subunit alpha type-1	0.839803
seq.5918.5	PSME1	Proteasome activator complex subunit 1	0.82326
seq.3867.49	SEZ6L2	Seizure 6-like protein 2	0.817225
seq.2612.5	EIF5	Eukaryotic translation initiation factor 5	0.813123
seq.6641.60	UBB	PolyUbiquitin K48-linked	0.811633
seq.4490.65	IL3RA	Interleukin-3 receptor subunit alpha	0.799696
seq.3875.62	ARID3A	AT-rich interactive domain-containing protein 3A	0.794092
seq.3874.8	UBE2L3	Ubiquitin-conjugating enzyme E2 L3	0.791656
seq.5900.11	HINT1	Histidine triad nucleotide-binding protein 1	0.790986
seq.2642.4	PAFAH1B2	Platelet-activating factor acetylhydrolase IB subunit beta	0.790673
seq.13090.17	S100A6	Protein S100-A6	0.789827
seq.4389.2	DHH	Desert hedgehog protein N-product	0.789067
seq.3860.7	PSMA6	Proteasome subunit alpha type-6	0.787806
seq.5882.34	EEF1B2	Elongation factor 1-beta	0.787544
seq.4393.3	FGF16	Fibroblast growth factor 16	0.785997
seq.3522.57	VIP	Vasoactive Intestinal Peptide	0.78308
seq.4130.71	FGF6	Fibroblast growth factor 6	0.779257
seq.3897.61	PDXP	Pyridoxal phosphate phosphatase	0.772063
seq.5356.2	MIF	Macrophage migration inhibitory factor	0.772037
seq.4394.71	FGF8	Fibroblast growth factor 8 isoform A	0.770155
seq.4396.54	IFNL1	Interferon lambda-1	0.769992
seq.2747.3	ULBP3	NKG2D ligand 3	0.768962
seq.2647.66	GDI2	Rab GDP dissociation inhibitor beta	0.763251

seq.4547.59	FLRT1	Leucine-rich repeat transmembrane protein FLRT1	0.759277
seq.3294.55	CFC1	Cryptic protein	0.756841
seq.2846.24	RPS27A	Ubiquitin+1, truncated mutation for UbB	0.75244
seq.4706.17	EPB41	Protein 4.1	0.751487
seq.3855.56	PRDX1	Peroxiredoxin-1	0.751113
seq.2998.53	JAM3	Junctional adhesion molecule C	0.750382
seq.5018.68	PRDX6	Peroxiredoxin-6	0.749395
seq.2970.60	AREG	Amphiregulin	0.747881
seq.14116.129	S100A4	Protein S100-A4	0.745317
seq.5225.50	CSNK2A1 CSNK2B	Casein kinase II 2-alpha:2-beta heterotetramer	0.743769
seq.6653.58	CD47	Leukocyte surface antigen CD47	0.743342
seq.3466.8	PRKACA	cAMP-dependent protein kinase catalytic subunit alpha	0.743151
seq.2634.2	IL2RG	Cytokine receptor common subunit gamma	0.738845
seq.3488.64	CAT	Catalase	0.73421
seq.4271.75	PFDN5	Prefoldin subunit 5	0.728961
seq.5870.23	BAD	Bcl2-associated agonist of cell death	0.721327
seq.3010.53	TSLP	Thymic stromal lymphopoietin	0.718335
seq.5226.36	CSNK2A2 CSNK2B	Casein kinase II 2-alpha':2-beta heterotetramer	0.71821
seq.3865.53	RPS6KA5	Ribosomal protein S6 kinase alpha-5	0.715461
seq.8458.111	SNCA	Alpha-synuclein	0.713262
seq.3893.64	MSLN	Mesothelin	0.712307
seq.4474.19	RPS27A	Ubiquitin	0.707435
seq.3397.7	PTPN11	Tyrosine-protein phosphatase non-receptor type 11	0.706904
seq.3839.60	AIP	AH receptor-interacting protein	0.701219

PC = principal component

Table S2A. Differentially expressed proteins significantly correlated with age in covariate-adjusted analysis at 24 hours post-injury. Bolded proteins are statistically significant at $p < 0.05$ and $q < 0.3$.

Aptamer Sequence ID	Entrez Gene Symbol	Target Full Name	Partial Correlation with Age	p-value	FDR q-value
seq.2844.53	**TIE1**	**Tyrosine-protein kinase receptor Tie-1, soluble**	0.8421	0.0006	0.2923
seq.2711.6	**CNTFR**	**Ciliary neurotrophic factor receptor subunit alpha**	0.8281	0.0009	0.2923
seq.3362.61	**CHRDL1**	**Chordin-like protein 1**	0.8144	0.0013	0.2923
seq.3303.23	**CST6**	**Cystatin-M**	0.8024	0.0017	0.2923
seq.3516.60	**CXCL12**	**Stromal cell-derived factor 1**	0.8010	0.0017	0.2923
seq.5364.7	**SET**	**Protein SET**	0.7999	0.0018	0.2923
seq.13109.82	**NEGR1**	**Neuronal growth regulator 1**	0.7810	0.0027	0.2923
seq.3601.54	**CHL1**	**Neural cell adhesion molecule L1-like protein**	0.7662	0.0037	0.2923
seq.5456.59	**CNDP1**	**Beta-Ala-His dipeptidase**	0.7602	0.0041	0.2923
seq.2744.57	**IGHG1**	**Immunoglobulin G**	0.7393	0.0060	0.2923
seq.2974.61	**CNTN1**	**Contactin-1**	0.7380	0.0061	0.2923
seq.3352.80	**CA6**	**Carbonic anhydrase 6**	0.7378	0.0062	0.2923
seq.14133.93	**IL1R2**	**Interleukin-1 receptor type 2**	0.7335	0.0066	0.2923
seq.4125.52	**AGER**	**Advanced glycosylation end product-specific receptor, soluble**	0.7235	0.0078	0.2923
seq.4162.54	**TF**	**Serotransferrin**	0.7181	0.0085	0.2923
seq.2816.50	**BCAM**	**Basal Cell Adhesion Molecule**	0.7166	0.0087	0.2923
seq.2677.1	**EGFR**	**Epidermal growth factor receptor**	0.7133	0.0092	0.2923
seq.13126.52	**DSC2**	**Desmocollin-2**	0.7016	0.0110	0.2923
seq.2960.66	**CFP**	**Properdin**	0.7001	0.0112	0.2923
seq.2827.23	**CX3CL1**	**Fractalkine**	0.6986	0.0115	0.2923

seq.5470.69	MED1	Mediator of RNA polymerase II transcription subunit 1	0.6905	0.0129	0.3024
seq.2638.12	CSF1R	Macrophage colony-stimulating factor 1 receptor	0.6873	0.0135	0.3024
seq.5036.50	TNFAIP6	Tumor necrosis factor-inducible gene 6 protein	0.6782	0.0154	0.3024
seq.5115.31	RELT	Tumor necrosis factor receptor superfamily member 19L	0.6775	0.0155	0.3024
seq.5105.2	RTN4R	Reticulon-4 receptor	0.6753	0.0160	0.3024
seq.3293.2	CD5L	CD5 antigen-like	0.6695	0.0173	0.3024
seq.3004.67	PDCD1LG2	Programmed cell death 1 ligand 2	0.6632	0.0187	0.3081
seq.5312.49	APOE	Apolipoprotein E (isoform E2)	0.6623	0.0189	0.3081
seq.11514.196	CD59	CD59 glycoprotein	0.6611	0.0192	0.3081
seq.4413.3	SLPI	Antileukoproteinase	0.6588	0.0198	0.3134
seq.4962.52	CDNF	Cerebral dopamine neurotrophic factor	0.6495	0.0223	0.3146
seq.5468.67	IL17RC	Interleukin-17 receptor C	0.6493	0.0223	0.3146
seq.2879.9	SERPINA3	Alpha-1-antichymotrypsin	0.6492	0.0224	0.3146
seq.14158.17	ANXA5	Annexin A5	0.6490	0.0224	0.3146
seq.4491.4	IL5RA	Interleukin-5 receptor subunit alpha	0.6485	0.0226	0.3146
seq.2828.82	SPINT1	Kunitz-type protease inhibitor 1	0.6482	0.0226	0.3146
seq.2819.23	CDH5	Cadherin-5	0.6450	0.0235	0.3146
seq.4324.33	CST2	Cystatin-SA	0.6400	0.0250	0.3182
seq.2789.26	MMP7	Matrilysin	0.6337	0.0269	0.3197
seq.2654.19	TNFRSF1A	Tumor necrosis factor receptor superfamily member 1A	0.6326	0.0273	0.3197
seq.4908.6	ENG	Endoglin	0.6323	0.0274	0.3197
seq.4157.2	F2	Thrombin	0.6316	0.0276	0.3197
seq.5392.73	FAS	Tumor necrosis factor receptor superfamily member 6	0.6298	0.0282	0.3236
seq.2620.4	IL6ST	Interleukin-6 receptor subunit beta	0.6273	0.0290	0.3255
seq.4982.54	PI3	Elafin	0.6232	0.0304	0.3342

seq.2837.3	MET	Hepatocyte growth factor receptor	0.6196	0.0316	0.3378
seq.4124.24	HSPA1A	Heat shock 70 kDa protein 1A	0.6186	0.0320	0.3378
seq.4866.59	NTRK2	BDNF/NT-3 growth factors receptor	0.6156	0.0331	0.3406
seq.3628.3	MAP2K2	Dual specificity mitogen-activated protein kinase kinase 2	0.6119	0.0345	0.3516
seq.4234.8	IL1RL1	Interleukin-1 receptor-like 1	0.6076	0.0361	0.3516
seq.3438.10	FSTL3	Follistatin-related protein 3	0.6072	0.0363	0.3516
seq.9171.11	CSRP3	Cysteine and glycine-rich protein 3	0.6069	0.0364	0.3516
seq.4998.50	JAK2	Tyrosine-protein kinase JAK2	0.6062	0.0367	0.3516
seq.5069.9	CD55	Complement decay-accelerating factor	0.6057	0.0369	0.3516
seq.3074.6	LBP	Lipopolysaccharide-binding protein	0.6017	0.0385	0.3516
seq.3607.71	DKK3	Dickkopf-related protein 3	0.6015	0.0386	0.3516
seq.14134.49	AMIGO2	Amphoterin-induced protein 2	0.6010	0.0388	0.3516
seq.9216.100	PLXNB2	Plexin-B2	0.6009	0.0388	0.3516
seq.3799.11	CA3	Carbonic anhydrase 3	0.5990	0.0396	0.3516
seq.5122.92	SEMA6A	Semaphorin-6A	0.5904	0.0433	0.3516
seq.3708.62	A2M	Alpha-2-macroglobulin	0.5903	0.0433	0.3516
seq.4534.10	PRSS22	Brain-specific serine protease 4	0.5892	0.0438	0.3516
seq.3034.1	GFAP	Glial fibrillary acidic protein	0.5884	0.0442	0.3516
seq.5121.3	SEMA6B	Semaphorin-6B	0.5882	0.0443	0.3516
seq.5487.7	SLAMF7	SLAM family member 7	0.5839	0.0462	0.3516
seq.3042.7	MB	Myoglobin	0.5834	0.0465	0.3516
seq.4471.50	TGM3	Protein-glutamine gamma-glutamyltransferase E	0.5804	0.0479	0.3516
seq.7648.9	MYBPC1	Myosin-binding protein C, slow-type	0.5800	0.0481	0.3516
seq.3581.53	AHSG	Alpha-2-HS-glycoprotein	0.5800	0.0481	0.3516
seq.8465.52	CTSH	Cathepsin H	0.5793	0.0484	0.3516
seq.2991.9	IL1R1	Interleukin-1 receptor type 1	0.5791	0.0485	0.3516
seq.2670.67	CKM	Creatine kinase M-type	0.5787	0.0487	0.3516

seq.2501.51	CDH1	Cadherin-1	0.5779	0.0491	0.3516
seq.11510.31	APOL1	Apolipoprotein L1	0.5770	0.0495	0.3516
seq.3322.52	LRIG3	Leucine-rich repeats and immunoglobulin-like domains protein 3	0.5763	0.0499	0.3516
seq.3657.74	PPP3R1	Calcineurin subunit B type 1	-0.5763	0.0498	0.3516
seq.3585.54	BSG	Basigin	-0.5773	0.0493	0.3516
seq.3313.21	FCN2	Ficolin-2	-0.5776	0.0492	0.3516
seq.2182.54	C4A	Complement C4b	-0.5787	0.0487	0.3516
seq.2891.1	CD22	B-cell receptor CD22	-0.5801	0.0480	0.3516
seq.3890.8	LDHB	L-lactate dehydrogenase B chain	-0.5836	0.0464	0.3516
seq.5360.9	AKT2	RAC-beta serine/threonine-protein kinase	-0.5843	0.0460	0.3516
seq.4355.13	DAPK2	Death-associated protein kinase 2	-0.5872	0.0447	0.3516
seq.3643.90	SLITRK1	SLIT and NTRK-like protein 1	-0.5873	0.0447	0.3516
seq.5383.14	TNFRSF13C	Tumor necrosis factor receptor superfamily member 13C	-0.5881	0.0443	0.3516
seq.13105.7	SNAP25	Synaptosomal-associated protein 25	-0.5906	0.0432	0.3516
seq.4548.4	FUT3	Galactoside 3(4)-L-fucosyltransferase	-0.5910	0.0430	0.3516
seq.4994.178	HNRNPK	Heterogeneous nuclear ribonucleoprotein K	-0.5914	0.0429	0.3516
seq.3391.10	PIK3CG	Phosphatidylinositol 4,5-bisphosphate 3-kinase catalytic subunit gamma isoform	-0.5922	0.0425	0.3516
seq.3043.49	SPARC	SPARC	-0.5928	0.0422	0.3516
seq.4132.27	FST	Follistatin	-0.5948	0.0414	0.3516
seq.4708.3	HSD17B1	Estradiol 17-beta-dehydrogenase 1	-0.5959	0.0409	0.3516
seq.4138.25	IL20	Interleukin-20	-0.5960	0.0409	0.3516
seq.3445.53	IL15RA	Interleukin-15 receptor subunit alpha	-0.5964	0.0407	0.3516
seq.3766.51	STX1A	Syntaxin-1A	-0.5991	0.0395	0.3516
seq.8480.29	EFEMP1	EGF-containing fibulin-like extracellular matrix protein 1	-0.5997	0.0393	0.3516
seq.4719.58	PDIA3	Protein disulfide-isomerase A3	-0.6007	0.0389	0.3516

seq.3217.74	SERPINE2	Glia-derived nexin	-0.6007	0.0389	0.3516
seq.3474.19	THBS1	Thrombospondin-1	-0.6049	0.0372	0.3516
seq.3292.75	CD48	CD48 antigen	-0.6067	0.0365	0.3516
seq.2976.58	DSG1	Desmoglein-1	-0.6076	0.0361	0.3516
seq.3285.23	C1R	Complement C1r subcomponent	-0.6076	0.0361	0.3516
seq.13089.6	HIF1A	Hypoxia-inducible factor 1-alpha	-0.6171	0.0326	0.3378
seq.4543.65	COL23A1	Collagen alpha-1(XXIII) chain	-0.6173	0.0325	0.3378
seq.4878.3	F10	Coagulation Factor X	-0.6179	0.0323	0.3378
seq.3434.34	FN1	Fibronectin Fragment 3	-0.6190	0.0318	0.3378
seq.2632.5	IL12RB1	Interleukin-12 receptor subunit beta-1	-0.6211	0.0311	0.3378
seq.3235.50	WFIKKN2	WAP, Kazal, immunoglobulin, Kunitz and NTR domain-containing protein 2	-0.6239	0.0301	0.3342
seq.4359.87	DYRK3	Dual specificity tyrosine-phosphorylation-regulated kinase 3	-0.6270	0.0291	0.3255
seq.5854.60	MAPT	Microtubule-associated protein tau	-0.6287	0.0285	0.3248
seq.3827.22	PAK6	Serine/threonine-protein kinase PAK 6	-0.6323	0.0274	0.3197
seq.3381.24	LYN	Tyrosine-protein kinase Lyn, isoform B	-0.6326	0.0273	0.3197
seq.3797.1	CDH2	Cadherin-2	-0.6356	0.0263	0.3197
seq.4452.9	LRRTM1	Leucine-rich repeat transmembrane neuronal protein 1	-0.6367	0.0260	0.3197
seq.2985.35	CXCL1	Growth-regulated alpha protein	-0.6381	0.0256	0.3187
seq.5093.47	JAG2	Protein jagged-2	-0.6391	0.0253	0.3182
seq.3644.5	DKKL1	Dickkopf-like protein 1	-0.6395	0.0251	0.3182
seq.3591.51	CDH6	Cadherin-6	-0.6403	0.0249	0.3182
seq.3037.62	IL1B	Interleukin-1 beta	-0.6403	0.0249	0.3182
seq.2783.18	CCL3L1	C-C motif chemokine 3-like 1	-0.6430	0.0241	0.3182
seq.3399.31	STAB2	Stabilin-2	-0.6450	0.0235	0.3146
seq.4718.5	PPIB	Peptidyl-prolyl cis-trans isomerase B	-0.6452	0.0235	0.3146
seq.10361.25	OAS1	2'-5'-oligoadenylate synthase 1	-0.6453	0.0235	0.3146
seq.2524.56	HMGB1	High mobility group protein B1	-0.6480	0.0227	0.3146

seq.5108.72	NOTCH3	Neurogenic locus notch homolog protein 3	-0.6489	0.0224	0.3146
seq.2640.3	MST1R	Macrophage-stimulating protein receptor	-0.6497	0.0222	0.3146
seq.3388.58	PAK7	Serine/threonine-protein kinase PAK 7	-0.6517	0.0217	0.3146
seq.2997.8	JAM2	Junctional adhesion molecule B	-0.6565	0.0204	0.3146
seq.5015.15	PLA2G7	Platelet-activating factor acetylhydrolase	-0.6616	0.0191	0.3081
seq.13124.20	ISLR2	Immunoglobulin superfamily containing leucine-rich repeat protein 2	-0.6627	0.0188	0.3081
seq.3647.49	TLR4	Toll-like receptor 4:Lymphocyte antigen 96 complex	-0.6672	0.0178	0.3034
seq.8463.2	SOD3	Extracellular superoxide dismutase [Cu-Zn]	-0.6672	0.0178	0.3034
seq.5026.66	RPS3	40S ribosomal protein S3	-0.6704	0.0170	0.3024
seq.3630.27	CADM3	Cell adhesion molecule 3	-0.6713	0.0168	0.3024
seq.4786.58	CMPK1	UMP-CMP kinase	-0.6715	0.0168	0.3024
seq.3437.80	FLT3	Receptor-type tyrosine-protein kinase FLT3	-0.6729	0.0165	0.3024
seq.5116.62	ROBO2	Roundabout homolog 2	-0.6737	0.0163	0.3024
seq.2381.52	C5	Complement C5	-0.6738	0.0163	0.3024
seq.3171.57	APP	Amyloid beta A4 protein	-0.6740	0.0162	0.3024
seq.3519.3	CCL17	C-C motif chemokine 17	-0.6772	0.0156	0.3024
seq.3640.14	LRPAP1	alpha-2-macroglobulin receptor-associated protein	-0.6806	0.0148	0.3024
seq.5410.53	CDH15	Cadherin-15	-0.6825	0.0145	0.3024
seq.4771.10	SMPDL3A	Acid sphingomyelinase-like phosphodiesterase 3a	-0.6826	0.0144	0.3024
seq.3873.51	TPO	Thyroid peroxidase	-0.6861	0.0138	0.3024
seq.3435.53	FN1	Fibronectin Fragment 4	-0.6867	0.0136	0.3024
seq.5262.57	BCAR3	Breast cancer anti-estrogen resistance protein 3	-0.6883	0.0133	0.3024
seq.3024.18	SERPINF2	Alpha-2-antiplasmin	-0.6891	0.0132	0.3024
seq.2943.5	CYP3A4	Cytochrome P450 3A4	-0.6913	0.0128	0.3024
seq.5013.2	CLIC1	Chloride intracellular channel protein 1	-0.6946	0.0122	0.3024

seq.3182.38	**ENTPD1**	**Ectonucleoside triphosphate diphosphohydrolase 1**	-0.6989	0.0114	0.2923
seq.2649.77	**ICAM3**	**Intercellular adhesion molecule 3**	-0.6995	0.0113	0.2923
seq.5062.60	**CD226**	**CD226 antigen**	-0.7033	0.0107	0.2923
seq.2474.54	**APCS**	**Serum amyloid P-component**	-0.7034	0.0107	0.2923
seq.3079.62	**RARRES2**	**Retinoic acid receptor responder protein 2**	-0.7059	0.0103	0.2923
seq.13093.6	**SECTM1**	**Secreted and transmembrane protein 1**	-0.7062	0.0103	0.2923
seq.9190.7	**CD63**	**CD63 antigen**	-0.7077	0.0100	0.2923
seq.13131.5	**HK1**	**Hexokinase-1**	-0.7086	0.0099	0.2923
seq.2946.52	**CFD**	**Complement factor D**	-0.7096	0.0097	0.2923
seq.4925.54	**MMP13**	**Collagenase 3**	-0.7112	0.0095	0.2923
seq.4906.35	**F5**	**Coagulation Factor V**	-0.7151	0.0090	0.2923
seq.5508.62	**CTSD**	**Cathepsin D**	-0.7159	0.0088	0.2923
seq.3472.40	**BIRC5**	**Baculoviral IAP repeat-containing protein 5**	-0.7202	0.0082	0.2923
seq.4567.82	**SH2D1A**	**SH2 domain-containing protein 1A**	-0.7203	0.0082	0.2923
seq.4482.66	**C5**	**Complement C5b-C6 complex**	-0.7244	0.0077	0.2923
seq.5259.2	**MAP3K7**	**Mitogen-activated protein kinase kinase kinase 7:TGF-beta-activated kinase 1 and MAP3K7-binding protein 1 fusion**	-0.7245	0.0077	0.2923
seq.3169.70	**IDUA**	**Alpha-L-iduronidase**	-0.7303	0.0070	0.2923
seq.10363.13	**SMAD3**	**Mothers against decapentaplegic homolog 3**	-0.7304	0.0070	0.2923
seq.2859.69	**HDAC8**	**Histone deacetylase 8**	-0.7362	0.0063	0.2923
seq.3629.60	**CDC42BPB**	**Serine/threonine-protein kinase MRCK beta**	-0.7412	0.0058	0.2923
seq.4891.50	**GCG**	**Glucagon**	-0.7461	0.0053	0.2923
seq.4693.72	**HIBADH**	**3-hydroxyisobutyrate dehydrogenase, mitochondrial**	-0.7482	0.0051	0.2923
seq.5261.13	**ABL2**	**Abelson tyrosine-protein kinase 2**	-0.7539	0.0046	0.2923
seq.13117.232	**CHKB**	**Choline/ethanolamine kinase**	-0.7607	0.0041	0.2923
seq.3605.77	**MASP1**	**Mannan-binding lectin serine protease 1**	-0.7655	0.0037	0.2923

seq.5328.33	**EGFR**	**Epidermal growth factor receptor variant III**	-0.7714	0.0033	0.2923
seq.5255.22	**PDE4D**	**cAMP-specific 3',5'-cyclic phosphodiesterase 4D**	-0.7730	0.0032	0.2923
seq.3323.37	**LRP8**	**Low-density lipoprotein receptor-related protein 8**	-0.7775	0.0029	0.2923
seq.3477.63	**NTRK1**	**High affinity nerve growth factor receptor**	-0.7779	0.0029	0.2923
seq.3624.3	**PRSS27**	**Serine protease 27**	-0.7829	0.0026	0.2923
seq.3508.78	**CCL22**	**C-C motif chemokine 22**	-0.7970	0.0019	0.2923

Table S2B. Differentially expressed proteins significantly correlated with age in covariate-adjusted analysis at 72-hours post-injury. Bolded proteins are statistically significant at $p < 0.05$ and $q < 0.3$.

Aptamer Sequence ID	Entrez Gene Symbol	Target Full Name	Partial Correlation with Age	p-value	FDR q-value
seq.3516.60	**CXCL12**	**Stromal cell-derived factor 1**	0.916599	2.76E-05	0.035785
seq.3362.61	**CHRDL1**	**Chordin-like protein 1**	0.888681	0.000111	0.072223
seq.4324.33	**CST2**	**Cystatin-SA**	0.832457	0.000779	0.202644
seq.3303.23	**CST6**	**Cystatin-M**	0.832361	0.000781	0.202644
seq.2571.12	**IGFBP3**	**Insulin-like growth factor-binding protein 3**	0.811697	0.001346	0.244509
seq.14131.37	**EFNB2**	**Ephrin-B2**	0.810999	0.00137	0.244509
seq.5115.31	**RELT**	**Tumor necrosis factor receptor superfamily member 19L**	0.804198	0.001615	0.244509
seq.4534.10	**PRSS22**	**Brain-specific serine protease 4**	0.802099	0.001697	0.244509
seq.3795.6	**ADAM9**	**Disintegrin and metalloproteinase domain-containing protein 9**	0.795358	0.001982	0.257036
seq.3628.3	**MAP2K2**	**Dual specificity mitogen-activated protein kinase kinase 2**	0.779522	0.002795	0.291166
seq.2609.59	**CST3**	**Cystatin-C**	0.777326	0.002926	0.291166
seq.2654.19	**TNFRSF1A**	**Tumor necrosis factor receptor superfamily member 1A**	0.771329	0.003305	0.291166
seq.3352.80	**CA6**	**Carbonic anhydrase 6**	0.770245	0.003378	0.291166
seq.2944.66	**NBL1**	**Neuroblastoma suppressor of tumorigenicity 1**	0.763034	0.00389	0.291166
seq.5036.50	**TNFAIP6**	**Tumor necrosis factor-inducible gene 6 protein**	0.755392	0.004496	0.291166
seq.4125.52	**AGER**	**Advanced glycosylation end product-specific receptor, soluble**	0.753832	0.004628	0.291166
seq.2900.53	**CCL14**	**C-C motif chemokine 14**	0.753686	0.004641	0.291166
seq.5364.7	**SET**	**Protein SET**	0.75283	0.004714	0.291166

seq.2789.26	**MMP7**	**Matrilysin**	0.748418	0.005109	0.299358
seq.2844.53	TIE1	**Tyrosine-protein kinase receptor Tie-1, soluble**	0.739882	0.005942	0.299358
seq.5349.69	DLL1	**Delta-like protein 1**	0.735329	0.006426	0.299358
seq.5121.3	SEMA6B	**Semaphorin-6B**	0.732416	0.006751	0.299358
seq.3320.49	IGFBP7	**Insulin-like growth factor-binding protein 7**	0.72813	0.007252	0.299358
seq.5456.59	CNDP1	Beta-Ala-His dipeptidase	0.721533	0.008076	0.308063
seq.4910.21	PLA2G1B	Phospholipase A2	0.708219	0.00995	0.343118
seq.3049.61	PRSS1	Trypsin-1	0.70286	0.010789	0.346184
seq.4491.4	IL5RA	Interleukin-5 receptor subunit alpha	0.700361	0.011197	0.346184
seq.3220.40	RET	Proto-oncogene tyrosine-protein kinase receptor Ret	0.700146	0.011233	0.346184
seq.5392.73	FAS	Tumor necrosis factor receptor superfamily member 6	0.697158	0.011738	0.346184
seq.3061.61	CTSB	Cathepsin B	0.697122	0.011744	0.346184
seq.3073.51	IL18BP	Interleukin-18-binding protein	0.690766	0.012874	0.366893
seq.3284.75	BGN	Biglycan	0.690018	0.013012	0.366893
seq.2615.60	EFNA5	Ephrin-A5	0.677269	0.015539	0.416186
seq.4867.15	VEGFA	Vascular endothelial growth factor A, isoform 121	0.670776	0.016956	0.416186
seq.5111.15	NRXN3	Neurexin-3-beta	0.670338	0.017055	0.416186
seq.13095.51	REG1A	Lithostathine-1-alpha	0.670231	0.017079	0.416186
seq.4500.50	CLEC11A	Stem cell growth factor-alpha	0.669138	0.017328	0.416186
seq.3059.50	TNFSF13B	Tumor necrosis factor ligand superfamily member 13B	0.665146	0.018259	0.420651
seq.5139.32	UNC5C	Netrin receptor UNC5C	0.664195	0.018487	0.420651
seq.4962.52	CDNF	Cerebral dopamine neurotrophic factor	0.661088	0.019243	0.424349
seq.4982.54	PI3	Elafin	0.65841	0.019914	0.424349
seq.2889.37	CTF1	Cardiotrophin-1	0.656544	0.020391	0.424349
seq.8479.4	MMP10	Stromelysin-2	0.655188	0.020743	0.424349

seq.3607.71	DKK3	Dickkopf-related protein 3	0.653636	0.021151	0.424349
seq.2748.3	INHBA	Inhibin beta A chain	0.651976	0.021594	0.424349
seq.13126.52	DSC2	Desmocollin-2	0.64691	0.022987	0.427536
seq.4413.3	SLPI	Antileukoproteinase	0.645988	0.023248	0.427536
seq.2666.53	DCN	Decorin	0.642636	0.024213	0.427536
seq.4922.13	CCL19	C-C motif chemokine 19	0.642405	0.024281	0.427536
seq.9211.19	SERPINF1	Pigment epithelium-derived factor	0.641385	0.024581	0.427536
seq.3495.15	CXCL6	C-X-C motif chemokine 6	0.640117	0.024957	0.427536
seq.5129.12	SCARF1	Scavenger receptor class F member 1	0.6398	0.025052	0.427536
seq.4908.6	ENG	Endoglin	0.636269	0.026126	0.434468
seq.5105.2	RTN4R	Reticulon-4 receptor	0.627854	0.028819	0.450792
seq.2966.65	CLEC11A	Stem Cell Growth Factor-beta	0.626995	0.029105	0.450792
seq.11514.196	CD59	CD59 glycoprotein	0.624961	0.02979	0.450792
seq.2436.49	CXCL16	C-X-C motif chemokine 16	0.624308	0.030012	0.450792
seq.3004.67	PDCD1LG2	Programmed cell death 1 ligand 2	0.622718	0.030558	0.450792
seq.2836.68	LCN2	Neutrophil gelatinase-associated lipocalin	0.622638	0.030586	0.450792
seq.11089.7	IGHA1	Immunoglobulin A	0.614417	0.033526	0.460434
seq.2816.50	BCAM	Basal Cell Adhesion Molecule	0.612432	0.034265	0.461446
seq.5357.60	NLGN4X	Neuroligin-4, X-linked	0.61178	0.034511	0.461446
seq.2711.6	CNTFR	Ciliary neurotrophic factor receptor subunit alpha	0.605395	0.03698	0.469097
seq.5459.33	CST1	Cystatin-SN	0.605018	0.03713	0.469097
seq.3152.57	TNFRSF1B	Tumor necrosis factor receptor superfamily member 1B	0.602687	0.038065	0.469097
seq.3805.16	ESM1	Endothelial cell-specific molecule 1	0.598261	0.039885	0.469097
seq.2843.13	SPINT2	Kunitz-type protease inhibitor 2	0.598149	0.039933	0.469097
seq.2753.2	C1QA	Complement C1q subcomponent	0.597461	0.040221	0.469097
seq.2788.55	MMP3	Stromelysin-1	0.595813	0.040919	0.469097
seq.5069.9	CD55	Complement decay-accelerating factor	0.594997	0.041268	0.469097

seq.5526.53	TNFRSF18	Tumor necrosis factor receptor superfamily member 18	0.594659	0.041413	0.469097
seq.3327.27	NTN4	Netrin-4	0.593736	0.041811	0.469097
seq.3440.7	GZMA	Granzyme A	0.592926	0.042162	0.469097
seq.3438.10	FSTL3	Follistatin-related protein 3	0.588455	0.044139	0.475162
seq.2828.82	SPINT1	Kunitz-type protease inhibitor 1	0.587223	0.044695	0.475162
seq.2913.1	CCL23	C-C motif chemokine 23	0.584817	0.045796	0.475312
seq.5090.49	LILRB1	Leukocyte immunoglobulin-like receptor subfamily B member 1	0.583996	0.046175	0.475312
seq.2781.63	CCL4L1	C-C motif chemokine 4-like	0.582112	0.047055	0.480558
seq.2981.9	ESAM	Endothelial cell-selective adhesion molecule	0.579973	0.048069	0.481379
seq.5487.7	SLAMF7	SLAM family member 7	0.577288	0.049363	0.481379
seq.3657.74	PPP3R1	Calcineurin subunit B type 1	-0.57629	0.049851	0.482516
seq.4543.65	COL23A1	Collagen alpha-1(XXIII) chain	-0.57738	0.049318	0.481379
seq.4428.1	CHST2	Carbohydrate sulfotransferase 2	-0.5778	0.049113	0.481379
seq.3196.6	HAPLN1	Hyaluronan and proteoglycan link protein 1	-0.57782	0.049107	0.481379
seq.3412.7	BCL2	Apoptosis regulator Bcl-2	-0.57919	0.048444	0.481379
seq.2746.56	CRLF2	Cytokine receptor-like factor 2	-0.58464	0.045875	0.475312
seq.2500.2	ANGPT4	Angiopoietin-4	-0.58533	0.045558	0.475312
seq.2762.30	FGF19	Fibroblast growth factor 19	-0.58768	0.044487	0.475162
seq.4449.67	GREM1	Gremlin-1	-0.58907	0.043864	0.475162
seq.3554.24	ADIPOQ	Adiponectin	-0.58952	0.043662	0.475162
seq.3296.92	CNTN2	Contactin-2	-0.59257	0.042316	0.469097
seq.13118.5	SMOC1	SPARC-related modular calcium-binding protein 1	-0.59463	0.041425	0.469097
seq.2192.63	CCL27	C-C motif chemokine 27	-0.59623	0.04074	0.469097
seq.4548.4	FUT3	Galactoside 3(4)-L-fucosyltransferase	-0.59628	0.04072	0.469097
seq.6641.60	UBB	PolyUbiquitin K48-linked	-0.60312	0.03789	0.469097
seq.4429.51	CHST6	Carbohydrate sulfotransferase 6	-0.60438	0.037386	0.469097
seq.4138.25	IL20	Interleukin-20	-0.60784	0.03602	0.467174

seq.3040.59	CCL3	C-C motif chemokine 3	-0.60844	0.035788	0.467174
seq.4703.87	LTA	Lymphotoxin-alpha	-0.61078	0.034889	0.461751
seq.9202.309	ATP5O	ATP synthase subunit O, mitochondrial	-0.61388	0.033725	0.460434
seq.4917.62	ITGAV	Integrin alpha-V: beta-5 complex	-0.61509	0.033278	0.460434
seq.2988.57	ICOS	Inducible T-cell costimulator	-0.61587	0.032994	0.460434
seq.7640.29	LRP1B	Low-density lipoprotein receptor-related protein 1B	-0.61901	0.031861	0.454112
seq.8469.41	IGFBP2	Insulin-like growth factor-binding protein 2	-0.61948	0.031693	0.454112
seq.3864.5	RPS7	40S ribosomal protein S7	-0.61977	0.031589	0.454112
seq.3323.37	LRP8	Low-density lipoprotein receptor-related protein 8	-0.62272	0.030556	0.450792
seq.5095.21	KIR2DL4	Killer cell immunoglobulin-like receptor 2DL4	-0.6281	0.028737	0.450792
seq.3235.50	WFIKKN2	WAP, Kazal, immunoglobulin, Kunitz and NTR domain-containing protein 2	-0.62917	0.028384	0.450792
seq.4144.13	CCL13	C-C motif chemokine 13	-0.63166	0.027577	0.450792
seq.4267.81	PES1	Pescadillo homolog	-0.63626	0.026128	0.434468
seq.3217.74	SERPINE2	Glia-derived nexin	-0.64503	0.023522	0.427536
seq.2945.25	ESR1	Estrogen receptor	-0.64734	0.022867	0.427536
seq.2190.55	F11	Coagulation Factor XI	-0.64942	0.02229	0.427536
seq.3594.6	CTSE	Cathepsin E	-0.65201	0.021584	0.424349
seq.3484.60	AGT	Angiotensinogen	-0.65223	0.021527	0.424349
seq.4551.72	ADGRG5	Adhesion G-protein coupled receptor G5	-0.65955	0.019626	0.424349
seq.2943.5	CYP3A4	Cytochrome P450 3A4	-0.66753	0.017699	0.417365
seq.9215.117	USP25	Ubiquitin carboxyl-terminal hydrolase 25	-0.67006	0.017118	0.416186
seq.5116.62	ROBO2	Roundabout homolog 2	-0.67041	0.017039	0.416186
seq.14123.34	C10orf54	Platelet receptor Gi24	-0.67398	0.016245	0.416186
seq.4566.24	RSPO2	R-spondin-2	-0.70077	0.01113	0.346184
seq.10361.25	OAS1	2'-5'-oligoadenylate synthase 1	-0.70754	0.010053	0.343118
seq.5062.60	CD226	CD226 antigen	-0.71155	0.009453	0.340553
seq.3827.22	PAK6	Serine/threonine-protein kinase PAK 6	-0.71266	0.009292	0.340553

seq.2839.2	TNFSF4	Tumor necrosis factor ligand superfamily member 4	-0.72301	0.007886	0.308063
seq.4706.17	**EPB41**	**Protein 4.1**	-0.72702	0.007386	0.299358
seq.4906.35	**F5**	**Coagulation Factor V**	-0.7298	0.007054	0.299358
seq.14151.4	**ISG15**	**Ubiquitin-like protein ISG15**	-0.73146	0.006861	0.299358
seq.5077.28	**EPHB2**	**Ephrin type-B receptor 2**	-0.73561	0.006396	0.299358
seq.3045.72	**PTN**	**Pleiotrophin**	-0.73565	0.006391	0.299358
seq.5255.22	**PDE4D**	**cAMP-specific 3',5'-cyclic phosphodiesterase 4D**	-0.74461	0.005469	0.299358
seq.4136.40	**IL-17D**	**Interleukin-17D**	-0.75322	0.004681	0.291166
seq.3640.14	**LRPAP1**	**alpha-2-macroglobulin receptor-associated protein**	-0.76329	0.003871	0.291166
seq.3427.63	**CSNK2A1**	**Casein kinase II subunit alpha**	-0.84341	0.000567	0.202644

Table S3. Fold-change and statistical significance of established traumatic brain injury biomarkers at 24- and 72-hours post-injury compared with healthy controls and between timepoints. Bolded values are statistically significant at $p < 0.05$ and $q < 0.3$.

Entrez Gene Symbol	Target Full Name	Fold-Change (24h vs. HC)	p-value (24h vs. HC)	Fold-Change (72h vs. HC)	p-value (72h vs. HC)	Fold-Change (72h vs. 24h)	p-value (72h vs. 24h)
UCHL1	Ubiquitin carboxyl-terminal hydrolase isozyme L1	1.0072	0.9643	1.0277	0.8646	1.0204	0.7830
BDNF	Brain-derived neurotrophic factor	-1.2979	0.0646	-1.4120	**0.0157**	-1.0879	0.2272
APCS	Serum amyloid P-component	-1.2779	0.0865	1.3086	0.0609	1.6723	**<0.0001**
KYNU	Kynureninase	1.6665	**0.0012**	1.3642	**0.0413**	-1.2216	**0.0112**
GFAP	Glial fibrillary acidic protein	4.9612	**0.0053**	5.7657	**0.0024**	1.1622	0.0853
NSE	Neuron-specific (gamma) enolase	2.1868	**0.0032**	1.5482	0.0903	-1.4125	**0.0002**

HC = healthy controls; 24h = 24 hours; 72h = 72 hours
